# Synthesis, Characterization, Antimicrobial Properties, and Antioxidant Activities of Silver-N-Heterocyclic Carbene Complexes

**DOI:** 10.1155/2023/3066299

**Published:** 2023-05-26

**Authors:** Donia Bensalah, Nevin Gurbuz, Ismail Özdemir, Rafik Gatri, Lamjed Mansour, Naceur Hamdi

**Affiliations:** ^1^Research Laboratory of Environmental Sciences and Technologies (LR16ES09), Higher Institute of Environmental Sciences and Technology, University of Carthage, Hammam-Lif, Tunisia; ^2^İnönü University, Faculty of Science and Art, Department of Chemistry, Malatya 44280, Turkey; ^3^İnönü University, Catalysis Research and Application Center, Malatya 44280, Turkey; ^4^Laboratory of Selective and Heterocyclic Organic Synthesis Biological Evaluation (LR17ES01), Faculty of Sciences of Tunis, University of Tunis El Manar Campus, Tunis 1092, Tunisia; ^5^Department of Zoology, College of Science, King Saud University, Riyadh, Saudi Arabia; ^6^Department of Chemistry, College of Science and Arts at Arras, Qassim University, P.O. Box 53, Arras 51921, Saudi Arabia

## Abstract

The emergence of antimicrobial resistance has become a major handicap in the fight against bacterial infections, prompting researchers to develop new, more effective, and multimodal alternatives. Silver and its complexes have long been used as antimicrobial agents in medicine because of their lack of resistance to silver, their low potency at low concentrations, and their low toxicity compared to most commonly used antibiotics. N-Heterocyclic carbenes (NHCs) are widely used for coordination of transition metals, mainly in catalytic chemistry. In this study, several N-alkylated benzimidazolium salts **2a**–**j** were synthesized. Then, the N-heterocyclic carbene (NHC) precursor was treated with Ag_2_O to give silver (I) NHC complexes (3a–j) at room temperature in dichloromethane for 48 h. Ten new silver-NHC complexes were fully characterized by nuclear magnetic resonance (NMR), Fourier transform infrared spectroscopy (FT-IR), elemental analysis, and LC-MSMS (for complexes) techniques. The antibacterial and antioxidant activities of salt **2** and its silver complex 3 were evaluated. All of these complexes were more effective against bacterial strains than comparable ligands. With MIC values ranging from 6.25 to 100 g/ml, the Ag-NHC complex effectively showed strong antibacterial activity. Antioxidant activity was also tested using conventional techniques, such as 2, 2-diphenyl-1-picrylhydrazine (DPPH) and hydrogen peroxide scavenging assays. In DPPH and ABTS experiments, compounds **3a**, **3b**, **3c**, **3e**, **3g,** and **3i** showed significant clearance.

## 1. Introduction

The world faces a perilous challenge known as antimicrobial resistance (AMR), the ability of microorganisms to withstand commonly used antibiotics [1]. This surge in AMR presents a significant risk to public health, leading to higher death rates, increased medical expenses, and reduced effectiveness of antimicrobial therapy [2]. The rise in AMR can be traced back to the misuse of antibiotics and the scarcity of new drugs to replace the currently compromised antimicrobial drugs [3]. In response to the issue of multiple treatment-resistant germs, the pharmaceutical and medical industries are actively seeking new, powerful, and less toxic antimicrobial drugs. The term antimicrobial resistance (AMR) refers to the capacity of microorganisms to withstand commonly-used antibiotics [1]. If left unchecked, this phenomenon can escalate morbidity, mortality, and healthcare expenditures [2, 3]. Currently, the heterocyclic compounds are the most sought-after components of effective anti-AMR medications [4–7]. The role of these compounds in the creation of antibacterial agents is highlighted through several key examples. Furthermore, metal-NHC complexes have been primarily utilized in catalytic chemistry [8, 9]. In recent times, NHC ligands have exhibited potential as carrier molecules for anticancer drugs [10]. Researchers continue to explore the vast potential of NHC-metal complexes, which is evident from the increasing number of published reviews [11, 12]. Combatting bacterial and cancerous infections could be aided with the application of Silver (I)-N-Heterocyclic Carbene (Ag(I)-NHC) complexes. Recent years have witnessed a surge in research regarding these compounds' potential for antimicrobial and anticancer applications [13, 14]. Creating effective antimicrobial Ag^−^NHC requires limiting the Ag^+^dissociation rate in affected regions. The structure of the NHC ligand holds sway over the activity of NHC-silver complex [15], with factors such as hydrophobic substitution and steric bulk on the imidazole ring causing a delay in silver ion release [16]. Tacke's group showed that the minimum inhibitory concentration (MIC) of the silver complex against various Gram-positive, Gram-negative, and mycobacteria ranged from 20 to 3.13 *μ*g/mL (35.3 to 5.52 *μ*M) [17]. Haque et al.published a comparative study in which a series of mononuclear and binuclear silver (I) complexes were synthesized [18–20]. Furthermore, silver carbene complexes show biological effects. [21] Gave an overview of this achievement, including the structural features and synthetic routes and uses of silver NHC complexes [22–24]. The presence of bulky electron-donating substituents attached to carbene ligands enhanced the antibacterial activity of silver complexes [23–28].

Here, we report the synthesis, characterization, and study of antibacterial and antifungal activities of ten novel asymmetric benzimidazole salts and their substituted NHC silver complexes. The characterization of these NHC-silver complexes is consistent with the proposed formula. Using the agar dilution method, the antimicrobial activity of these compounds was examined against Gram (+)/(−) bacterial and fungal strains. In antibacterial studies it was observed that the NHC-silver complex was more active against fungal strains than against Gram-positive and Gram-negative bacterial strains. In addition, the antioxidant properties of these compounds were also investigated.

## 2. Experimental

### 2.1. Materials and Methods

All manipulations were carried out in air. All chemicals and solvents were purchased from Sigma-Aldrich and Merck. The solvents such as dimethylformamide (DMF), dichloromethane, and diethyl ether were purified by distillation over the drying agents. Melting points were determined with an Electrothermal-9200 melting points apparatus. The elemental analysis measurements were determined by LECO CHNS-932 elemental analyser. Fourier transform infrared spectra were obtained in the range 450–4000 cm^−1^ on a Perkin Elmer Spectrum 100 Spectrophotometer. The mass analysis was determined by using a Thermo Scientific Exactive Plus Benchtop Full-Scan Orbitrap Mass Spectrometer LC-MS/MS analyzer. ^1^H NMR and ^13^C NMR spectra were recorded at 400 MHz (1H), 100 MHz (^13^C) in CDCl_3_ with tetramethylsilane as an internal reference (Malaty, Turkey). The NMR studies were carried out in high-quality 5 mm NMR tubes. Signals are quoted in parts per million as *δ* downfield from tetramethylsilane (*δ* = 0.00) as an internal standard. NMR multiplicities are abbreviated as follows: *s* = singlet, *d* = doublet, *t* = triplet, and *m* = multiplet. In the investigation of antimicrobial properties of silver-NHCs, some microorganisms defined in the American Type Culture Collection (ATCC) were preferred. Mueller–Hinton Broth was purchased from HiMedia Laboratories Pvt. Ltd. (Mumbai, India), and RPMI 1640 broth was purchased from Sigma-Aldrich (Chemie GmbH, Taufkirchen, Germany). The spectroscopic data of the new silver-NHCs are presented as follows.

### 2.2. Preparation of Benzimidazolium Salts **2a**-**j**

1-(2-morpholinoethyl)-5,6-dimethylbenzimidazole (1 mmol) was reacted with various alkyl chlorides/bromides (1.04 mmol) in toluene (10 mL) at 80°C for 72 hours to give Benzimidazole salt. Addition of diethyl ether (15 ml) gave a white solid which was then filtered off. After washing with diethyl ether (3 × 15 mL), the solid was dried in vacuo.

#### 2.2.1. 1-(2-Morpholinoethyl)-3-(2,3,5,6-Tetramethylbenzyl)-5,6-Dimethylbenzimidazolium Chloride 2a

Yield: 90%; Mp 256°C; *ν* (CN) = 1556 cm^−1^; HR-AM (H-ESI II) analysis calculated (*m*/*z*) for cationic part of [C_26_H_36_N_3_O]^+^: 406.59; found (*m*/*z*): 406.2801. ^1^H NMR (400 MHz, CDCl_3_, 25) *δ* (ppm) = 2.25 (d, 12H, CH_3(c,d,e,f)_), 2.37 (s, 3H, CH_3(b)_), 2.41 (s, 3H, CH_3(a)_), 2.47 (s, 4H, H_4′,8′_), 2.73 (s, 2H, H_2′_), 3.40 (s, 4H, H_5′,7′_), 4.82 (s, 2H, H_1′_), 5.63 (s, 2H, H_1″_), 7.08 (s, 1H, H_5″_); 7.24 (s, 1H, H_4_), 7.45 (s, 1H, H_7_), 10.16 (s, 1H, H_2_); ^13^C NMR (100 MHz, CDCl_3_) *δ* (ppm) = 16.22 (C_c,f_), 20.68 (C_a_), 20.77 (C_b_), 20.90 (C_d,e_), 44.03 (C_1′_), 46.66 (C_1″_), 53.34 (C_4′,8′_), 56.10 (C_2′_), 66.84 (C_5′,7′_), 112.74 (C_4_), 113.01 (C_7_), 127.96 (C_5″_), 129.85 (C_8,9_), 130.00 (C_4″,6″_),133.68 (C_3″,7″_), 134.10 (C_6_), 135.28 (C_5_), 137.04 (C_2″_), and 142.43 (C_2_).

#### 2.2.2. 1-(2-Morpholinoethyl)-3-(2,3,4,5,6-Pentamethylbenzyl)-5,6-Dimethylbenzimidazolium Chloride 2b

Yield: 83%; Mp 250°C; *ν* (CN) = 1557 cm^−1^; HR-AM (H-ESI II) analysis calculated (*m*/*z*) for cationic part of [C_27_H_38_N_3_O]^+^: 420.62; found (*m*/*z*): 420.2951. ^1^H NMR (400 MHz, CDCl_3_) *δ* (ppm) = 2.24 (s, 6H, CH_3(c,g)_), 2.26 (s, 3H, CH_3(e)_), 2.29 (s, 6H, CH_3(d,f)_), 2.38 (s, 3H, CH_3(b)_), 2.42 (s, 3H, CH_3(a)_), 2.45 (s, 4H, H_4′,8′_), 2.71 (s, 2H, H_2′_), 3.36 (s, 4H, H_5′,7′_), 4.83 (s, 2H, H_1′_); 5.61 (s, 2H, H_1″_), 7.31 (s, 1H, H_4_), 7.47 (s, 1H, H_7_), 9.94 (s, 1H, H_2_); ^13^C NMR (100 MHz, CDCl_3_) *δ* (ppm) = 17.09 (C_c,g_), 17.20 (C_e_), 17.39 (C_d,e_), 20.76 (C_a_), 20.88 (C_b_), 44.05 (C_1′_), 47.02 (C_1″_), 53.30 (C_4′,8′_), 56.12 (C_2′_), 66.74 (C_5′,7′_), 112.80 (C_4_), 112.91 (C_7_), 125.25 (C_5″_), 129.79 (C_8_), 130.06 (C_9_), 133.57 (C_4″,6″_), 134.09 (C_3″,7″_), 134.09 (C_6_), 137.01 (C_5_), 137.50 (C_2″_), and 142.16 (C_2_).

#### 2.2.3. 3-(Cyclohexylmethyl)-1-(2-Morpholinoethyl)-5,6-Dimethylbenzimidazolium Bromide 2c

Yield: 46%; Mp 253°C; *ν* (CN) = 1565 cm^−1^; HR-AM (H-ESI II) analysis calculated (*m*/*z*) for cationic part of [C_22_H_34_N_3_O]^+^: 356.54; found (*m*/*z*): 356.2649. ^1^H NMR (400 MHz, CDCl_3_) *δ* (ppm) = 1.08–1.26 (m, 6H, H_4″,5″,6″_), 1.63 (d, 2H, H_7″_), 1.74 (d, 2H, H_3″_), 1.96–2.00 (m, 1H, H_2″_), 2.45 (s, 6H, CH_3(a,b)_), 2.68 (s, 4H, H_4′,8′_), 2.99 (t, 2H, H_2′_), 3.65 (t, 4H, H_5′,7′_), 4.32 (d, 2H, H_1′_); 4.81 (t, 2H, H_1″_), 7.39 (s, 1H, H_4_), 7.55 (s, 1H, H_7_), 11.00 (s, 1H, H_2_); ^13^C NMR (100 MHz, CDCl_3_) *δ* (ppm) = 20.79 (C_a,b_), 20.83 (C_4″,6″_), 25.41 (C_5″_), 25.84 (C_2″_), 30.40 (C_3″_), 37.71 (C_7″_), 43.61 (C_1′_), 53.14 (C_4′,8′_), 53.40 (C_2′_), 55.92 (C_1″_), 66.64 (C_5′,7′_), 112.81 (C_4_), 112.93 (C_7_), 129.64 (C_8_), 129.92 (C_9_), 137.31 (C_6_), 137.41 (C_5_), and 142.60 (C_2_).

#### 2.2.4. 3-(Cyclobutylmethyl)-1-(2-Morpholinoethyl)-5,6-Dimethylbenzimidazolium Bromide 2d

Yield: 72%; Mp 219°C; *ν* (CN) = 1562 cm^−1^; HR-AM (H-ESI II) analysis calculated (*m*/*z*) for cationic part of [C_20_H_30_N_3_O]^+^: 328.48; found (*m*/*z*): 328.2341. ^1^H NMR (400 MHz, CDCl_3_) *δ* (ppm) = 1.90–1.98 (m, 4H, H_3″,5″_), 2.11–2.16 (m, 2H, H_4″_), 2.45 (s, 6H, CH_3(a,b)_), 2.69 (s, 4H, H_4′,8′_), 3.00–3.06 (m, 2H, H_2″_), 3.65 (s, 4H, H_5′,7′_), 4.49 (d, 2H, H_1′_), 4.81 (t, 2H, H_1″_), 7.41 (s, 1H, H_4_); 7.54 (s, 1H, H_7_), 11.03 (s, 1H, H_2_), ^13^C NMR (100 MHz, CDCl_3_) *δ* (ppm) = 18.07 (C_4″_), 20.81 (C_a,b_), 25.85 (C_3″,5″_), 34.39 (C_2″_), 43.71 (C_1′_), 52.02 (C_4′,8′_), 53.43 (C_2′_), 56.04 (C_1″_), 66.67 (C_5′,7′_), 112.80 (C_4_), 112.84 (C_7_), 129.76 (C_8_), 129.80 (C_9_), 137.32 (C_6_), 137.45 (C_5_), and 142.07 (C_2_).

#### 2.2.5. 3-(4-Chlorobenzyl)-1-(2-Morpholinoethyl)-5,6-Dimethylbenzimidazolium Chloride 2e

Yield: 73%; Mp 244°C; *ν* (CN) = 1563 cm^−1^; HR-AM (H-ESI II) analysis calculated (*m*/*z*) for cationic part of [C_22_H_27_ClN_3_O]^+^: 384.93; found (*m*/*z*): 384.1787. ^1^H NMR (400 MHz, CDCl_3_) *δ* (ppm) = 2.38 (d, 6H, CH_3(a,b)_), 2.59 (s, 4H, H_4v,8′_), 2.93 (s, 2H, H_2'_), 3.62 (s, 4H, H_5′,7′_), 4.67 (s, 2H, H_1'_), 5.80 (s, 2H, H_1″_), 7.31 (t, 3H, H_4,3″,7″_); 7.46 (d, 3H, H_7,4″,6″_), 11.61 (s, 1H, H_2_); ^13^C NMR (100 MHz, CDCl_3_) *δ* (ppm) = 20.80 (C_b_), 20.82 (C_a_), 44.13 (C_1′_), 50.24 (C_4′,8′_), 53.44 (C_2'_), 56.09 (C_1″_), 66.86 (C_5′,7′_), 112.76 (C_4_), 113.23 (C_7_), 129.46 (C_8,9_), 129.57 (C_3″,7″_), 129.74 (C_6_),129.91 (C_5_), 131.88 (C_4″,6″_), 135.25 (C_5″_), 137.48 (C_2″_), and 143.27 (C_2_).

#### 2.2.6. 3-Benzyl-1-(2-Morpholinoethyl)-5,6-Dimethylbenzimidazolium Chloride 2f

Yield: 77%; Mp 244°C; *ν* (CN) = 1566 cm^−1^; HR-AM (H-ESI II) analysis calculated (*m*/*z*) for cationic part of [C_22_H_28_N_3_O]^+^: 350.49; found (*m*/*z*): 350.2181. ^1^H NMR (400 MHz, CDCl_3_) *δ* (ppm) = 2.38 (d, 6H, CH_3(a,b)_), 2.56 (s, 4H, H_4′,8′_), 2.90 (s, 2H, H_2′_), 3.59 (s, 4H, H_5′,7′_), 4.69 (s, 2H, H_1′_), 5.75 (s, 2H, H_1″_), 7.33 (t, 4H, H_4,4″,5″,6″_); 7.46 (d, 3H, H_3″,7″,7_), 11.57 (s, 1H, H_2_); ^13^C NMR (100 MHz, CDCl_3_) *δ* (ppm) = 20.77 (C_a,b_), 44.25 (C_1'_), 50.97 (C_4′,8′_), 53.50 (C_2′_), 56.19 (C_1″_), 67.00 (C_5′,7′_), 112.71 (C_4_), 113.33 (C_7_), 128.16 (C_5″_), 129.20 (C_4″,6″_), 129.40 (C_8,9_), 129.63 (C_3″,7″_),,129.93 (C_6_), 133.35 (C_5_), 137.26 (C_2″_), and 143.30 (C_2_).

#### 2.2.7. 3-(2-Methoxyethyl)-1-(2-Morpholinoethyl)-5,6-Dimethylbenzimidazolium Chloride 2g

Yield: 91%; Mp 160°C; *ν* (CN) = 1566 cm^−1^; HR-AM (H-ESI II) analysis calculated (*m*/*z*) for cationic part of [C_18_H_28_N_3_O_2_]^+^: 318.44; found (*m*/*z*): 318.2121. ^1^H NMR (400 MHz, CDCl_3_) *δ* (ppm) = 2.42 (s, 6H, CH_3(a,b)_), 2.72 (s, 4H, H_4′,8′_), 3.07(s, 2H, H_2′_), 3.32 (s, 3H, CH_3(4″)_), 3.70 (s, 4H, H_5′,7′_), 3.90 (t, 2H, H_2″_), 4.68 (s, 2H, H_1′_), 4.80 (s, 2H, H_1″_), 7.48 (s, 1H, H_4_); 7.54 (s, 1H, H_7_), 11.06 (s, 1H, H_2_); ^13^C NMR (100 MHz, CDCl_3_) *δ* (ppm) = 20.74 (C_a,b_), 47.47 (C_1′_), 53.24 (C_2′,4′,8′_), 59.18 (C_1′,4″_), 70.21 (C_2″,5′,7′_), 112.63 (C_7_), 113.26 (C_8_), 113.44 (C_9_), 129.63 (C_4_), 130.40 (C_6_), 137.18 (C_5_), and 142.89 (C_2_).

#### 2.2.8. 3-(2-Ethoxyethyl)-1-(2-Morpholinoethyl)-5,6-Dimethylbenzimidazolium Chloride 2h

Yield: 92%; Mp 109°C; *ν* (CN) = 1563 cm^−1^; HR-AM (H-ESI II) analysis calculated (*m*/*z*) for cationic part of [C_19_H_28_N_3_O_2_]^+^: 332.47; found (*m*/*z*): 332.2278. ^1^H NMR (400 MHz, CDCl_3_) *δ* (ppm) = 1.09 (t, 3H, CH_3(5″)_), 2.42 (d, 6H, CH_3(a,b)_), 2.84 (s, 4H, H_4′,8′_), 3.21 (s, 2H, H_2′_), 3.49 (q, 2H, H_4″_), 3.76 (s, 4H, H_5′,7′_), 3.92 (t, 2H, H_2″_), 4.67 (t, 2H, H_1'_), 4.91 (s, 2H, H_1″_), 7.53 (s, 1H, H_4_); 7.63 (s, 1H, H_7_), 11.08 (s, 1H, H_2_); ^13^C NMR (100 MHz, CDCl_3_) *δ* (ppm) = 14.87 (C_5″_), 20.50 (C_b_), 20.56 (C_a_), 47.74(C_1′_), 52.90 (C_2′,4′,8′_), 66.73 (C_1″_), 68.10 (C_5′,7′,2″,4″_), 112.49 (C_7_), 113.65 (C_4_), 129.48 (C_8,9_), 130.30 (C_6_), 136.95 (C_5_), and 142.51 (C_2_).

#### 2.2.9. 3-(3,5-Di-Tert-Butylbenzyl)-1-(2-Morpholinoethyl)-5,6-Dimethylbenzimidazolium Bromide 2i

Yield: 92%; Mp 247°C; *ν* (CN) = 1564 cm^−1^; HR-AM (H-ESI II) analysis calculated (*m*/*z*) for cationic part of [C_30_H_44_N_3_O]^+^: 462.71; found (*m*/*z*): 462.3415. ^1^H NMR (400 MHz, CDCl_3_) *δ* (ppm) = 1.28 (s, 18H, CH_3(c,d,e,f,g,h)_), 2.37 (s, 3H, CH_3(b)_), 2.42 (s, 3H, CH_3(a)_), 2.57 (s, 4H, H_4′,8′_), 2.93 (s, 2H, H_2′_), 3.56 (s, 4H, H_5′,7′_), 4.72 (t, 2H, H_1′_), 5.68 (s, 2H, H_1″_), 7.29 (d, 2H, H_3″,7″_); 7.36 (s, 1H, H_4_), 7.41 (s, 1H, H_5″_), 7.46 (s, 1H, H_7_), 11.17 (s, 1H, H_2_); ^13^C NMR (100 MHz, CDCl_3_) *δ* (ppm) = 20.79 (C_b_), 20.81 (C_a_), 31.50 (C_c,d,e,f,g,h_), 35.07 (C_8″,9″_), 52.14 (C_1'_), 53.44 (C_2′,4′,8″_), 65.97 (C_1″,5′,7′_), 112.86 (C_4_), 113.57 (C_7_), 122.84 (C_5″_), 123.42 (C_4″,6″_), 129.76 (C_8_),130.14 (C_9_), 131.93 (C_3″,7″_), 137.20 (C_5,6_), 142.36 (C_2″_), and 152.29 (C_2_).

#### 2.2.10. 3-(4-(Tert-Butyl)Benzyl)-1-(2-Morpholinoethyl)-5,6-Dimethylbenzimidazolium Bromide 2j

Yield: 80%; Mp 224°C; *ν* (CN) = 1557 cm^−1^; HR-AM (H-ESI II) analysis calculated (*m*/*z*) for cationic part of [C_26_H_36_N_3_O]^+^: 406.60; found (*m*/*z*): 406.2800. ^1^H NMR (400 MHz, CDCl_3_) *δ* (ppm) = 1.26 (s, 9H, CH_3(c,d,e)_), 2.40 (d, 6H, CH_3(a,b)_), 2.68 (s, 4H, H_4′,8′_), 3.02 (s, 2H, H_2′_), 3.62 (s, 4H, H_5′,7′_), 4.81 (s, 2H, H_1'_), 5.66 (s, 2H, H_1″_), 7.40 (q, 5H, H_4,3″,4″,6″,7″_), 7.56 (s, 1H, H_7_); 11.08 (s, 1H, H_2_); ^13^C NMR (100 MHz, CDCl_3_) *δ* (ppm) = 20.78 (C_b_), 20.82 (C_a_), 31.27 (C_c,d,e_), 34.78 (C_8″_), 50.78 (C_1′_), 53.26 (C_4′,8′,2′_), 55.73 (C_1″_), 66.50 (C_5′,7″_), 112.87 (C_4_), 113.25 (C_7_), 126.43 (C_5″_), 128.12 (C_4″,6″_), 129.62 (C_8_),129.86 (C_9_), 129.99 (C_3″,7″_), 137.43 (C_5,6_), 142.34 (C_2″_), and 152.59 (C_2_).

#### 2.2.11. General Procedure for the Preparation of Silver(I)–NHC Complexes **3a**-**j**

Benzimidazolium salt (1.0 mmol) (**2a-j**) and Ag_2_O (1.5 mmol) were dissolved in 15 mL of dichloromethane and stirred at room temperature in the dark for 48 hours. Under reduced pressure, the solvent was extracted from the reaction mixture after filtration through celite.

#### 2.2.12. Chloro [1-(2-Morpholinoethyl)-3-(2,3,5,6-Tetramethylbenzyl)-5,6-Dimethylbenzimidazole-2-Ylidene] Silver 3a

Yield: 78%; Mp 236°C; *ν* (CN) = 1442 cm^−1^; HR-AM (H-ESI II) analysis calculated (*m*/*z*) for cationic part of [C_26_H_35_N_3_O]^+^: 405.59; found (*m*/*z*): 406.2800. ^1^H NMR (400 MHz, CDCl_3_) *δ* (ppm) = 2.13 (s, 6H, CH_3(c,f)_), 2.29 (s, 6H, CH_3(d,e)_), 2.41 (d, 6H, CH_3(a,b)_), 2.47 (t, 4H, H_4′,8′_), 2.72 (t, 2H, H_2′_), 3.62 (t, 4H, H_5′,7′_), 4.38 (t, 2H, H_1'_), 5.38 (s, 2H, H_1″_), 7.14 (s, 1H, H_5″_); 7.24 (s, 1H, H_4_), 7.25 (s, 1H, H_7_); ^13^C NMR (100 MHz, CDCl_3_) *δ* (ppm) = 16.27 (C_c, f_), 20.61 (C_b_), 20.65 (C_a_), 20.84 (C_d,e_), 46.99 (C_1′_), 47.65 (C_1″_), 54.03 (C_4′,8′_), 57.97 (C_2′_), 67.01 (C_5′,7′_), 111.67 (C_4_), 111.70 (C_7_), 130.07 (C_5″_), 132.23 (C_4″,6″_), 133.13 (C_8,9_), 133.22 (C_5_), 133.52 (C_6_), 133.78 (C_3″,7″_), and 135.46 (C_2″_). Anal. Calcd for C_26_H_35_AgClN_3_O: C, 56.89%; H, 6.43%; N, 7.66%. Found: C, 56.9; H, 6.5; N, 7.7%.

#### 2.2.13. Chloro [1-(2-Morpholinoethyl)-3-(2,3,4,5,6-Pentamethylbenzyl)-5,6-Dimethylbenzimidazole-2-Ylidene] Silver 3b

Yield: 77%; Mp 243°C; *ν* (CN) = 1443 cm^−1^; HR-AM (H-ESI II) analysis calculated (*m*/*z*) for cationic part of [C_27_H_37_N_3_O]^+^: 419.62; found (*m*/*z*): 420.2951. ^1^H NMR (400 MHz, CDCl_3_) *δ* (ppm) = 2.18 (d, 6H, CH_3(c,g)_), 2.28 (s, 6H, CH_3(d,f)_), 2.33 (s, 3H, CH_3(e)_), 2.42 (s, 6H, CH_3(a,b)_), 2.47 (t, 4H, H_4′,8′_), 2.71 (t, 2H, CH3_2′_), 3.62 (t, 4H, H_5′,7′_), 4.37 (t, 2H, H_1′_), 5.37 (s, 2H, H_1″_), 7.23 (s, 1H, H_4_), 7.30 (s, 1H, H_7_); ^13^C NMR (100 MHz, CDCl_3_) *δ* (ppm) = 17.22 (C_c,g_), 17.29 (C_e_), 17.53 (C_d,f_), 20.61 (C_b_), 20.63 (C_a_), 47.33 (C_1′_), 47.68 (C_1″_), 54.03 (C_4′,8′_), 58.02 (C_2′_), 67.02 (C_5′,7′_), 111.68 (C_4_), 126.90 (C_7_), 132.38 (C_5_), 132.99 (C_8,9_), 133.14 (C_4″,6″_), 133.44 (C_6_), 133.75 (C_5_), 134.35 (C_3″,7″_), and 137.40 (C_2″_). Anal. Calcd for C_27_H_37_AgClN_3_O: C, 57.61%; H, 6.63%; N, 7.46%. Found: C, 57.7; H, 6.7; N, 7.5%.

#### 2.2.14. Bromo [3-(Cyclohexylmethyl)-1-(2-Morpholinoethyl)-5,6-Dimethylbenzimidazole-2-Ylidene] Silver 3c

Yield: 72%; Mp 227°C; *ν* (CN) = 1443 cm^−1^; HR-AM (H-ESI II) analysis calculated (*m*/*z*) for cationic part of [C_22_H_33_N_3_O]^+^: 355.54; found (*m*/*z*): 356.2648. ^1^H NMR (400 MHz, CDCl_3_) *δ* (ppm) = 1.09–1.22 (m, 6H, H_4″,5″,6″_), 1.59–1.66 (m, 2H, H_3″_), 1.72 (d, 2H, H_7″_), 1.89–195 (m, 1H, H_2″_), 2.40 (d, 6H, CH_3(a,b)_), 2.51 (t, 4H, H_4′,8′_), 2.77 (t, 2H, H_2′_), 3.68 (t, 4H, H_5′,7′_), 4.15 (d, 2H, H_1″_); 4.42 (t, 2H, H_1′_), 7.20 (s, 1H, H_4_), 7.23 (s, 1H, H_7_); ^13^C NMR (100 MHz, CDCl_3_) *δ* (ppm) = 20.59 (Ca,b), 25.70 (C_4″,6″_), 26.12 (C_5″_), 31.13 (C_3″,7″_), 38.29 (C_2″_), 46.90 (C_1′_), 54.15 (C_4′,8′_), 55.70 (C_1″_), 58.09 (C_2′_),67.04 (C_5′,7′_), 111.66 (C_4_),112.11 (C_7_), 132.28 (C_8_), 132.64 (C_9_), and 133.57 (C_5,6_). Anal. Calcd for C_22_H_33_AgBrN_3_O: C, 48.64%; H, 6.12%; N, 7.73%. Found: C, 48.7; H, 6.3; N, 7.8%.

#### 2.2.15. Bromo [3-(Cyclobutylmethyl)-1-(2-Morpholinoethyl)-1-(2-Morpholinoethyl)-5,6-Dimethylbenzimidazole-2-Ylidene] Silver 3d

Yield: 70%; Mp 73°C; *ν* (CN) = 1446 cm^−1^; HR-AM (H-ESI II) analysis calculated (*m*/*z*) for cationic part of [C_20_H_29_N_3_O]^+^: 327.48; found (*m*/*z*): 328.2340. ^1^H NMR (400 MHz, CDCl_3_) *δ* (ppm) = 1.86–2.00 (m, 7H, H_2″,3″,4″,5″_), 2.39 (s, 6H, CH_3 (a, b)_) 2.50 (t, 4H, H_4′,8′_), 2.76 (t, 2H, H_2′_), 3.68 (t, 4H, H_5′,7′_), 4.33 (d, 2H, H_1″_), 4.41 (t, 2H, H_1′_), 7.22 (s, 2H, H_4,7_), ^13^C NMR (100 MHz, CDCl_3_) *δ* (ppm) = 18.25 (C_4″_), 20.55 (C_a,b_), 26.31 (C_3″,5″_), 35.66 (C_2″_), 46.92 (C_1′_), 53.56 (C_4′,8′_), 54.12 (C_2′_), 54.34 (C_1″_), 58.14 (C_5′_), 67.00 (C_7′_), 111.63 (C_4_), 111.94 (C_7_), 132.29 (C_8_), 132.39 (C_9_), and 133.55 (C_5,6_). Anal. Calcd for C_20_H_29_AgBrN_3_O: C, 46.62%; H, 5.67%; N, 8.16%. Found: C, 46.7; H, 5.37; N, 8.2%.

#### 2.2.16. Chloro [3-(4-Chlorobenzyl)-1-(2-Morpholinoethyl)-5,6-Dimethylbenzimidazole-2-Ylidene] Silver 3e

Yield: 80%; Mp 227°C; *ν* (CN) = 1441 cm^−1^; HR-AM (H-ESI II) analysis calculated (*m*/*z*) for cationic part of [C_22_H_26_ClN_3_O]^+^: 383.93; found (*m*/*z*): 384.1787. ^1^H NMR (400 MHz, CDCl_3_) *δ* (ppm) = 2.29 (s, 3H, CH_3(a)_), 2.35 (s, 3H, CH_3(b)_), 2.50 (t, 4H, H_4′,8′_), 2.77 (t, 2H, H_2′_), 3.66 (t, 4H, H_5′,7′_), 4.45 (t, 2H, H_1′_), 5.50 (s, 2H, H_1″_), 7.00 (s, 1H, H_4_); 7.14 (d, 2H, H_3″,7″_), 7.23 (t, 3H, H_7,4″,6″_); ^13^C NMR (100 MHz, CDCl_3_) *δ* (ppm) = 20.57 (C_b_), 20.59 (C_a_), 47.08 (C_1′_), 52.60 (C_1″_), 54.17 (C_4′,8′_), 58.14 (C_2′_), 67.01 (C_5′,7′_), 111.79 (C_4_), 112.22 (C_7_), 128.42 (C_8_), 129.38 (C_9_), 132.10 (C_3″,7″_),132.56 (C_4″,6″_), 133.84 (C_6_), 134.03 (C_5_), 134.09 (C_5″_), and 134.47 (C_2″_). Anal. Calcd for C_22_H_26_AgCl_2_N_3_O: C, 50.12%; H, 4.97%; N, 7.97%. Found: C, 50.3; H, 5.1; N, 8.1%.

#### 2.2.17. Chloro [3-Benzyl-1-(2-Morpholinoethyl)-5,6-Dimethylbenzimidazole-2-Ylidene] Silver 3f

Yield: 70%; Mp 214°C; *ν* (CN) = 1440 cm^−1^; HR-AM (H-ESI II) analysis calculated (*m*/*z*) for cationic part of [C_22_H_27_N_3_O]^+^: 349.49; found (*m*/*z*): 350.2180. ^1^H NMR (400 MHz, CDCl_3_) *δ* (ppm) = 2.31 (s, 3H, CH_3(b)_), 2.38 (s, 3H, CH_3(a)_), 2.52 (t, 4H, H_4′,8′_), 2.80 (t, 2H, H_2′_), 3.69 (t, 4H, H_5′,7′_), 4.47 (t, 2H, H_1′_), 5.55 (s, 2H, H_1″_), 7.09 (s, 1H, H_5″_); 7.21–7.24 (m, 3H, H_4,4″,6″_), 7.30 (t, 3H, H_3″,7″,7_); ^13^C NMR (100 MHz, CDCl_3_) *δ* (ppm) = 20.55 (C_a_), 20.59 (C_b_), 47.11 (C_1′_), 53.25 (C_1″_), 54.18 (C_4′,8′_), 58.12 (C_2′_), 67.03 (C_5′,7′_), 111.68 (C_4_), 112.40 (C_7_), 127.07 (C_5″_), 128.57 (C_4″,6″_), 129.18 (C_3″,7″_), 132.33 (C_8_),132.52(C_9_), 133.86 (C_5_), 133.93 (C_6_), and 135.29 (C_2″_).

Anal. Calcd for C_22_H_27_AgClN_3_O: C, 53.62%; H, 5.52%; N, 8.53%. Found: C, 53.7; H, 5.6; N, 8.6%.

#### 2.2.18. Chloro [3-(2-Methoxyethyl)-1-(2-Morpholinoethyl)-5,6-Dimethylbenzimidazole-2-Ylidene] Silver 3g

Yield: 75%; Mp 114°C; *ν* (CN) = 1434 cm^−1^; HR-AM (H-ESI II) analysis calculated (*m*/*z*) for cationic part of [C_18_H_27_N_3_O_2_]^+^: 317.44; found (*m*/*z*): 318.2134. ^1^H NMR (400 MHz, CDCl_3_) *δ* (ppm) = 2.39 (s, 6H, CH_3(a, b)_), 2.52 (t, 4H, H_4′,8′_), 2.78 (t, 2H, H_2′_), 3.29 (s, 3H, CH_3(4″)_), 3.68 (t, 4H, H_5′,7′_), 3.78 (t, 2H, H_2″_), 4.44 (t, 2H, H_1″_), 4.50 (t, 2H, H_1′_), 7.21 (s, 1H, H_4_); 7.30 (s, 1H, H_7_); ^13^C NMR (100 MHz, CDCl_3_) *δ* (ppm) = 20.55 (C_a, b_), 47.07 (C_1′_), 49.26 (C_1″_), 54.13 (C_4′,8′_), 58.20 (C_2′_), 59.23 (C_4″_), 66.98 (C_5′,7′_), 71.93 (C_2″_), 111.47(C_4_), 112.38 (C_7_), 132.16 (C_8_), 133.01 (C_9_), 133.58 (C_5_), and 133.61 (C_6_). Anal. Calcd for C_18_H_27_AgClN_3_O_2_: C, 46.92%; H, 5.91%; N, 9.12%. Found: C, 46.9; H, 6.1; N, 9.2%.

#### 2.2.19. Chloro [3-(2-Ethoxyethyl)-1-(2-Morpholinoethyl)-5,6-Dimethylbenzimidazole-2-Ylidene] Silver 3h

Yield: 74%; Mp 250°C; *ν* (CN) = 1444 cm^−1^; HR-AM (H-ESI II) analysis calculated (*m*/*z*) for cationic part of [C_19_H_27_N_3_O_2_]^+^: 331.47; found (*m*/*z*): 332.2290. ^1^H NMR (400 MHz, CDCl_3_) *δ* (ppm) = 1.10 (t, 3H, CH_3(5″)_), 2.39 (d, 6H, CH_3(a,b)_), 2.51 (t, 4H, H_4′,8′_), 2.77 (t, 2H, H_2′_), 3.44 (q, 2H, H_4″_), 3.68 (t, 4H, H_5′,7′_), 3.79 (t, 2H, H_2″_), 4.42 (t, 2H, H_1″_), 4.48 (t, 2H, H_1′_), 7.21 (s, 1H, H_4_); 7.34 (s, 1H, H_7_); ^13^C NMR (100 MHz, CDCl_3_) *δ* (ppm) = 15.15 (CH_3(5″)_), 20.46 (C_b_), 20.54 (C_a_), 47.03 (C_1′_), 49.57 (C_1″_), 54.14 (C_4′,8′_), 58.24 (C_2′_), 66.92 (C_4″_), 66.98 (C_5′,7′_), 69.81 (C_2″_), 111.40 (C_4_), 112.68 (C_7_), 132.18 (C_8_), 133.02 (C_9_), 133.52 (C_5_), and 133.56 (C_6_). Anal. Calcd for C_19_H_29_AgClN_3_O_2_: C, 48.07%; H, 6.16%; N, 8.85%. Found: C, 48.1; H, 6.12; N, 8.9%.

#### 2.2.20. Bromo [3-(3,5-Di-Tert-Butylbenzyl)-1-(2-Morpholinoethyl)-5,6-Dimethylbenzimidazole-2-ylidene] Silver 3i

Yield: 78%; Mp 177°C; *ν* (CN) = 1449 cm^−1^; HR-AM (H-ESI II) analysis calculated (*m*/*z*) for cationic part of [C_30_H_43_N_3_O]^+^: 461.71; found (*m*/*z*): 462.3418. ^1^H NMR (400 MHz, CDCl_3_) *δ* (ppm) = 1.26 (s, 18H, CH_3(c,d,e,f,g,h)_), 2.33 (s, 3H, CH_3(b)_), 2.38 (s, 3H, CH_3(a)_), 2.52 (t, 4H, H_4′,8′_), 2.80 (t, 2H, H_2′_), 3.68 (t, 4H, H_5′,7′_), 4.46 (t, 2H, H_1′_), 5.50 (s, 2H, H_1″_), 7.12 (d, 2H, H_3″,7″_); 7.18 (s, 1H, H_5_), 7.23 (s, 1H, H_4_), 7.35 (t, 1H, H_7_); ^13^C NMR (100 MHz, CDCl_3_) *δ* (ppm) = 20.50 (C_b_), 20.58 (C_a_), 31.49 (C_c,d,e,f,g,h_), 34.96 (C_8″,9″_), 47.06 (C_1′_), 53.95 (C_1″_), 54.18 (C_4′,8′_), 58.40 (C_2′_), 66.99 (C_5′,7′_), 111.62 (C_4_),112.59 (C_7_), 121.75 (C_5″_), 122.51 (C_4″,6_), 132.46 (C_8_), 132.57 (C_9_), 133.59 (C_5_), 133.62 (C_6_), 134.33 (C_3″,7″_), and 151.68 (C_2″_). Anal. Calcd for C_30_H_43_AgBrN_3_O: C, 55.48%; H, 6.67%; N, 6.47%. Found: C, 55.5; H, 6.7; N, 6.5%.

#### 2.2.21. Bromo [3-(4-(Tert-Butyl)B enzyl)-1-(2-Morpholinoethyl)-5,6-Dimethylbenzimidazole-2-Ylidene] Silver 3j

Yield: 76%; Mp 101°C; *ν* (CN) = 1446 cm^−1^; HR-AM (H-ESI II) analysis calculated (*m*/*z*) for cationic part of [C_26_H_35_N_3_O]^+^: 405.60; found (*m*/*z*): 406.2797. ^1^H NMR (400 MHz, CDCl_3_) *δ* (ppm) = 1.27 (s, 9H, CH_3(c,d,e)_), 2.33 (s, 3H, CH_3(a)_), 2.38 (s, 3H, CH_3(b)_), 2.52 (t, 4H, H_4′,8′_), 2.80 (t, 2H, H_2′_), 3.68 (t, 4H, H_5′,7′_), 4.47 (t, 2H, H_1′_), 5.50 (s, 2H, H_1″_), 7.15 (d, 2H, H_3″,7″_), 7.18 (s, 1H, H_4_); 7.23 (s, 1H, H_7_); 7.32 (d, 2H, H_4″,6″_); ^13^C NMR (100 MHz, CDCl_3_) *δ* (ppm) = 20.54 (C_b_), 20.57 (C_a_), 31.36 (C_c,d,e_), 34.69 (C_8″_), 47.08 (C_1′_), 52.83 (C_1″_), 54.16 (C_4′,8′_), 58.12 (C_2′_), 67.02 (C_5′,7′_), 111.63 (C_4_), 112.41 (C_7_),126.06 (C_5″_), 126.87 (C_4″,6″_), 132.36 (C_8_), 132.42 (C_9_), 132.49 (C_3″,7″_), 133.73 (C_5_), 133.80 (C_6_), and 151.57 (C_2″_). Anal. Calcd for C_26_H_35_AgBrN_3_O: C, 52.63%; H, 5.95%; N, 7.08%. Found: C, 52.7; H, 6.1; N, 7.1%.

### 2.3. Determination of Minimum Inhibitory Concentration of the Silver-Complexes **3a**-**j**

The antimicrobial activity of silver-NHCs complexes **3** was reported in terms of the minimum inhibitory concentration (MIC), according to previous work [29–32].

### 2.4. Antioxidant Activity

Antioxidant activity was expressed as EC50 (the concentration that causes 50% of effect). The free radical scavenging activity of the synthesized compounds was assessed by 2.2-diphenyl-1-picrylhydrazyl (DPPH) and 2.2′-azino-bis(3-ethylbenzothiazoline-6-sulfonic acid) (ABTS) radical assays. Butylated hydroxytoluene (BHT) was used as a control, and it is well known as a strong antioxidant compound.

#### 2.4.1. (DPPH) Radical Scavenging Activity

The DPPH assay is the simplest and most widely reported method for screening antioxidant activity dimethylsulfoxide (DMSO)/water (1/9; *v*/*v*) and diluted with ultrapure water to different concentrations (1, 0.5, 0.250, 0.125, 0.0625, 0.03125 mg·mL^−1^) [33, 34].

#### 2.4.2. (ABTS) Radical Scavenging Activity

This manipulation was carried out according to the protocol proposed by Re et al. [35] with some modifications.

## 3. Result and Discussion

### 3.1. Synthesis and Spectral Characterization

**Scheme 1:**
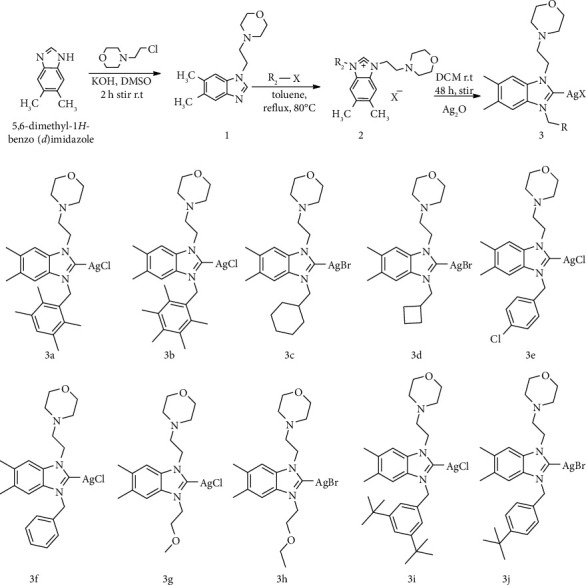
Synthesis of N-alkylated benzimidazole (1), benzimidazolium salts (2), and Ag(I)-NHC complexes (3).

NHC precursors and silver (I)-NHC complexes were successfully synthesized using a modified procedure [36]. After deprotonation in a basic medium, the first step is by equimolar amounts of 5,6-dimethylbenzimidazole and 4-(2-chloroethyl) morpholine in DMSO in the presence of KOH at room temperature the N-alkylation was carried out under reaction for 2 hours. To generate a single product upon formation of the NHC ligand, a second alkylation was performed at 80°C using 1 equivalent of some alkyl halide in refluxing toluene for 48 h. Following thin layer chromatography, the reactions were observed to form salts (2a-j) for each of the target chemicals. The reaction was monitored by thin layer chromatography, after which salt formation (2a-j) was observed for each target compound. The benzimidazolium salts (2a-j) are stable to air and moisture in both solid and solution states. The following protocol can be used to prepare silver-NHC complexes. Treat the free NHC with the correct silver source; (ii) treat the azole salt with an alkaline silver source such as Ag_2_O, AgCO_3_ and Ag(OAc); and (iii) treat the azole salt with a silver salt in the presence of an external base. Today, a popular and readily available method for the synthesis of silver-NHC complexes is the in situ deprotonation of azole salts using AgO as the main silver source. Therefore, in this work, silver NHC complexes (**3a-3j**) were prepared by in situ deprotonation method. The reaction was carried out at room temperature for 24 hours under dark conditions in the presence of dichloromethane as solvent. The target complex was obtained as an air-stable white solid in 70% to 80% yield. Silver complexes **3a**-**3j** are soluble in most organic solvents such as CH_2_Cl_2_, CHCl_3_, EtOAc, and DMSO, with the exception of nonpolar solvents such as n-pentane, n-hexane, and Et_2_O Scheme 1.

#### 3.1.1. FTIR Spectroscopy

Bands in N-alkylated benzimidazoles (1) and salts (2) are attributable to Caliph-Nbenzimi stretching vibrations [37, 38] and sometimes appear broad due to signal overlap with residual water molecules. The signal at 2875 cm^−1^ is caused by the CH stretching vibration of the aromatic ring. Although the Khalifa H stretching vibration of the alkyl chain is thought to be responsible for the appearance of signals in the 2875–2963 cm^−1^ range in the spectrum of salt 2, these signals in the silver **3** complex were found to be due to the coordination of the Ag(I) ion weakening and electron-donating properties of the alkyl group. For complex **3**, the CN stretching vibration occurs in a specific four-finger mode (f. fs) [39, 40]. The CN stretching vibration of the benzimidazole ring of salt 2 appears at 1356–1539 cm^−1^, while complex **3** does not appear in this mode. The CH vibrations of aromatic compounds occur at 729–901 cm^−1^.

The synthesis of salt **2** was confirmed by observing specific signals for alkyl protons with chemical shifts of 0–6 ppm and aromatic protons in the range of 6–8 ppm. In addition, new signals of the most deshielded protons and carbons (NCHN) appear between 9 and 12 ppm between 140 and 145 ppm in 13C NMR and facilitate the synthesis of NHC ligands 2 confirmed [41]. During silver metallization, the NCHN proton resonance of [42] NHC salts disappeared at 9–12 ppm, which may indicate Ag-NHC bonding. The aliphatic-CH_2_ protons of the benzyl substituents of the benzimidazolium salts were observed to be between *δ* = 5.43–5.50 ppm. In addition, CH_2_ proton singlets of morpholine were found at *δ* = 2.40 ppm and 3.60 ppm.

**Figure 1:**
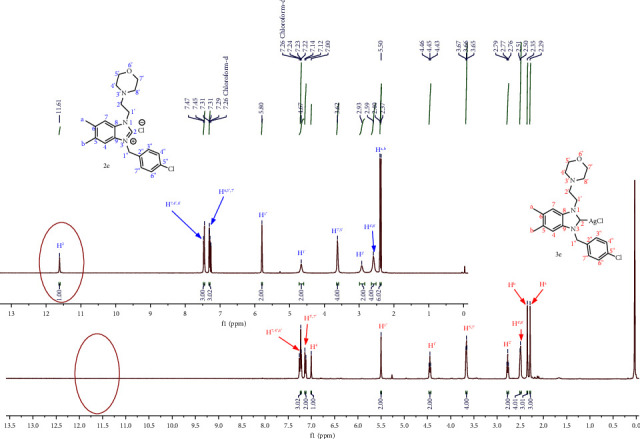
^1^H NMR spectrum of silver-carbene complex **3e** (in CDCl_3_, 400 MHz, 25°C, TMS).

**Figure 2:**
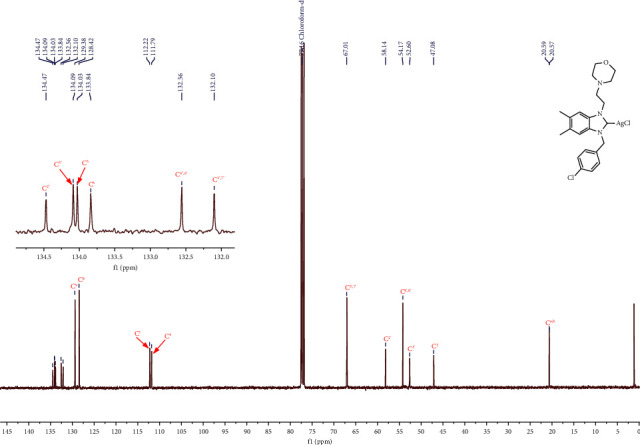
^13^C NMR spectrum of silver-carbene complex **3e** (in CDCl_3_, 100 MHz, 25°C, TMS).

In the ^13^C NMR spectrum of complex **3** of all silver NHCs, the characteristic signal of the C(2) carbon of benzimidazolium salt has completely disappeared, and the characteristic AgC(carbene) bond resonance of complex **3** was not observed, which is also the case in the literature mentioned and given as the reason for the fluctuating behavior of silver NHC [43]. This can be attributed to the dynamic behavior of the silver complex in solution and the poor relaxation of the carbine quarter carbon. For complexes **3a**-**3j**, aliphatic-CH_2_ carbon resonances of benzyl substituents were detected between *δ* = 47.08–52.6 ppm. In addition, the CH_2_ carbon resonance of morpholine was detected between *δ* = 52.6–67.01 ppm. The elemental analysis data of the silver complex are also consistent with the expected structure. Figures 1 and 2.

Table 1 summarizes some physical and spectroscopic data for the novel carbene-silver complex **3**.

**Figure 3:**
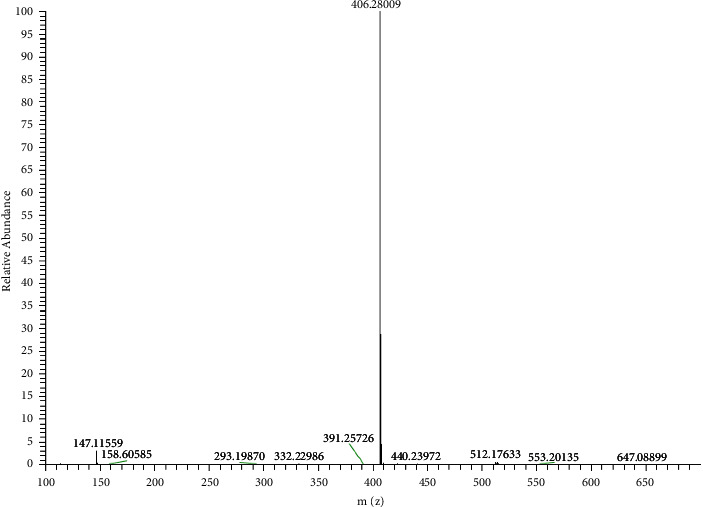
LC–MS/MS spectrum of silver-carbene complex **3a**.

Silver NHCs exhibit multiple structural modes both in the solid state and in solution. The properties of NHC ligands, temperature, solvent, and counterions are also affected by the silver NHC structure [43, 45]. However, it is often not possible to determine the structure of silver-NHC complexes in solution due to the presence of different species that participate in rapid equilibration at ambient temperature. However, it is often impossible to determine the structure of silver-NHC complexes in solution due to the presence of different species that participate in rapid equilibration at room temperature. Ligand exchange equilibria between neutral monocarbene complexes [AgX(NHC)] or ion pairs [Ag(NHC)_2_] + [AgX_2_] were demonstrated by variable temperature NMR studies using the association mechanism. To better understand the structure of our complexes, many attempts have been made to generate suitable silver complex crystals using solvent diffusion methods using different solvent systems including CH_2_Cl_2_/Et_2_O and EtOH/Et_2_O. Despite all efforts, it was not possible to isolate single crystals suitable for X-ray examination from the silver complexes. However, in the absence of crystallographic data, mass spectroscopy can be used to elucidate the structure of silver-NHC complexes. To understand the behavior of these complexes in solvent, only one of the complexes **3**, was studied by mass spectrometer LC/MS/MS. For this reason, complex **3a** was chosen as the model complex for mass analysis.  Figure 3.

**Figure 4:**
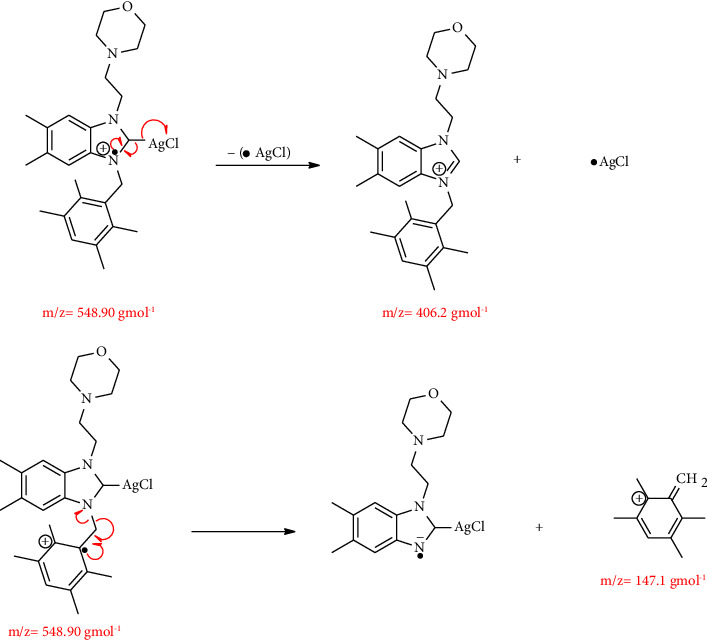
Mechanism of the fragmentation leading to the *m*/*z* = 406.28 peak.

The fragmentation leading to the *m*/*z* = 406.28 can occur via the mechanism of fragmentation given in Figure 4.

Complex 3a's LC-MS/MS spectra in the presence of chloroform as the solvent revealed that complex **3a** exists in the solution as [Ag(NHC)_2_] and [AgCl_2_]. Based on these results, we can infer that the structure of synthetic silver-NHC complexes is [Ag(NHC)_2_] + [AgX_2_] inside the solvent. Several silver-NHC structures with charge-balanced [Ag(NHC)_2_] +  cations and halogeno anions of type [AgX_2_] have been reported in the literature [46].

### 3.2. Antimicrobial Properties of Carbene-Based Silver-Complexes **3a**-**j**

**Figure 5:**
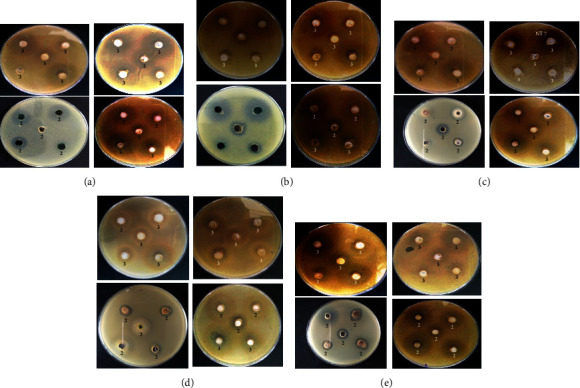
Antibacterial activities of synthetic products against bacteria stains. (a) Antibacterial activities of synthetic products against *Escherichia coli* (ATCC 25988). (b) Antibacterial activities of synthetic products against *Pseudomonas aeruginosa* (ATCC 27853). (c) Antibacterial activities of synthetic products against *Klebsilla pneumonia* (ATCC 700603). (d) Antibacterial activities of synthetic products against *Staphylococcus aureus* (ATCC 29213). (e) Antibacterial activities of synthetic products against *Methicillin-resistant Staphylococcus aureus* (ATCC 43300).

**Figure 6:**
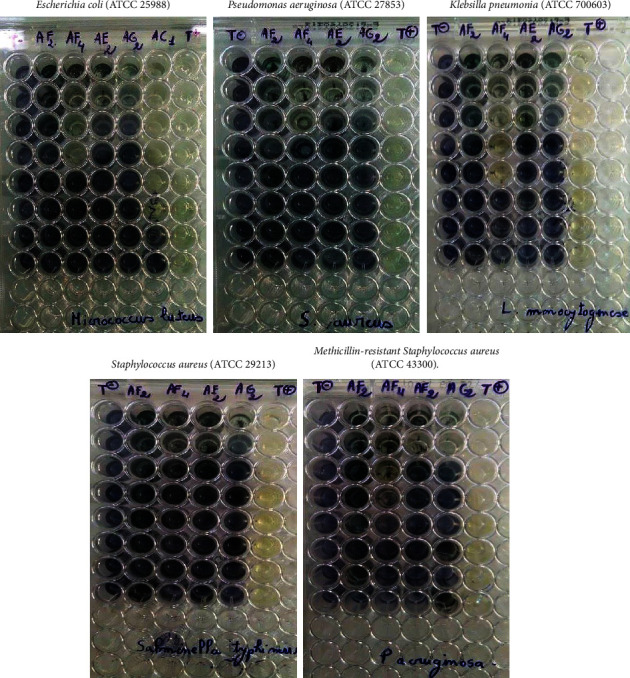
CMI against differents bacteria strains.

The minimum inhibition concentration (MIC), which is the lowest concentration of test sample that completely inhibits the growth of microorganisms, was determined for the antibacterial study by the broth dilution method and the disc diffusion method, respectively [47]. Zones of inhibition against *Escherichia coli* (ATCC 25988), *Pseudomonas aeruginosa* (ATCC 27853), *Klebsilla pneumonia* (ATCC 700603), *Staphylococcus aureus* (ATCC 29213), and *Methicillin-resistant Staphylococcus aureus* (ATCC 43300) were measured, and the minimum inhibitory concentrations of test samples were determine.  Figure 5 The MIC values of compounds **2**-**3** against all bacterial strains are tabulated in Table 2. (Figure 6)

As shown in Table 2, all silver NHCs inhibited the growth of bacterial and fungal strains. First, when we evaluated the antibacterial activity of silver NHC against Gram-negative bacterial strains, we could say that the **3a**, **3c,** and **3e** complexes showed the same activity against Escherichia coli (ATCC 25988) bacteria. The **3e**, **3h,** and **3i** complexes showed the least activity against Pseudomonas aeruginosa (ATCC 27853) bacteria. The **3c** complex showed lower activity against Klebsiella pneumoniae (ATCC 700603) bacteria. Of all the silver complexes, complexes **3b** and **3a** are the most sterically demanding and most active against Gram-negative bacterial strains. Likewise, all silver-NHC complexes were less active than ampicillin against *Staphylococcus aureus* (ATCC 29213). Promising results can be said to have been obtained when the antibacterial activity of all silver-NHC complexes was evaluated compared with standard drugs such as ampicillin and flucarbazole.

If we evaluate the antibacterial activity of silver NHCs against fungal strains, it can be said that some silver NHCs are less active than fluconazole, an antifungal drug used for many fungal infections. For example, all complexes except **2e** and **2g** showed the same anti-*C. albicans* (ATCC 14053) activity as fluconazole. Finally, silver NHC showed less or equal inhibitory activity against Gram-negative bacterial strains compared to Gram-positive bacterial strains and fungal strains. These results can be attributed to the outer membrane of Gram-negative bacteria making them more resistant to antimicrobial agents. These results suggest that *N* atom substitution of benzimidazol-2-ylidene ligands plays an important role in the antibacterial activity.

The antibacterial activity of NHC salts **2** showed lower minimal inhibitory concentration when compared to that of silver (I)-NHC complexes **3**. Interestingly, the silver-ligand bonding of complexes **3** resulted in promising activity against all bacterial strains, similar to that of ciprofloxacin. This could be due to the synergistic effects of silver-ligand bonding, which enhances the lipophilicity of complexes. It has been previously demonstrated that the silver-ligand bond is the critical factor for antibacterial activity, over other factors like degree of polymerization, chirality, or solubility of these complexes. In addition, the Ag(I)-NHC complexes displayed a broad antibacterial spectrum, potentially caused by ligand exchange phenomenon with S-(thiols).

Direct interaction occurs between silver ions and biological ligands, such as membranes, proteins, DNA and enzymes, while N- or O-donors at potential target points, such as bacterial sulfur containing proteins and enzymes, play a significant role [48]. The antimicrobial potential of silver complexes depends on the ease of the ligand exchange process that enables release of Ag^+^ ions, with the NHC ligands in silver (I)-NHC complexes only acting as carriers of silver ions to target sites in biological systems [49, 50]. Antimicrobial activity is influenced not only by the nature of silver complexes but also by the type of bacterial strains present, making it difficult to draw clear conclusions about the structure activity relationship that displays antimicrobial potential [51]. However, Table 2 indicates that the sensitivity of *B*. cereus to silver compounds is apparent from the larger zones of inhibition and smaller MIC values [52].

In addition, the presence of the benzimidazole aromatic ring increases lipophilicity and activity, which helps silver ions to pass through the cell membrane and enter the cell, destroying the function of organelles, resulting in obstruction of the respiratory system and respiratory system. Metabolic mechanisms of microorganisms [26, 53]. The cellular functions of microorganisms are affected by the interaction of silver ions with cellular proteins and DNA, which interact with thiol groups of various enzymes, causing them to denature. The activity of silver complexes on bacteria and fungi is closely related to their solubility, stability, lipophilicity and release rate of Ag^+^ ions. In previous studies, the authors reported increased antibacterial activity due to the lipophilicity of the silver-NHC complex and the increased release rate of Ag^+^ ions [27, 54]. For this reason, we here attempted to use different N-substituents on the NHC backbone to compare the effect of N-substituents on stability and lipophilicity. In addition, we believe that ether functional substituents can increase water solubility, so silver NHC may be more effective in antibacterial detection.

#### 3.2.1. Antioxidant Activity

**Figure 7:**
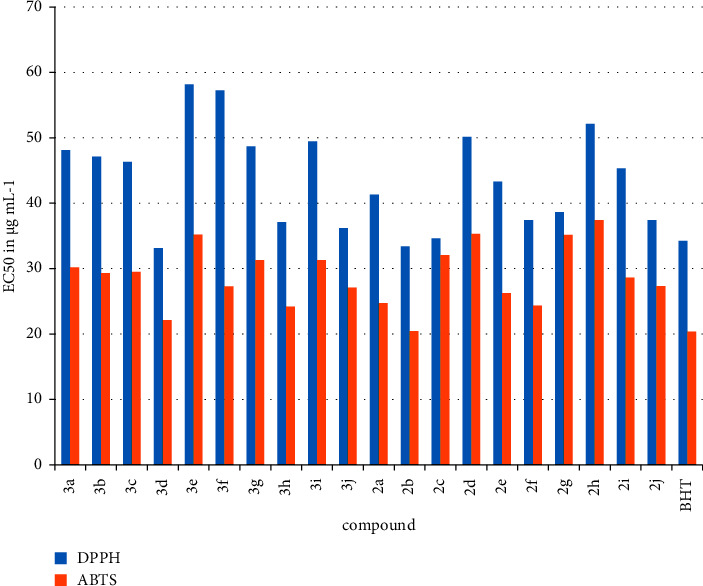
Antioxydant activity of the synthesized compounds **2a-j** et **3a-j** assessed by DPPH and ABTS techniques and expressed as EC50 in *μg*·mL^−1^. The BHT was used as control.

DPPH (1,1-diphenyl-2-picrylhydrazyl) antiradical assay and ABTS [2,20-azino-bis (3-ethylbenzothiazoline-6-sulfonic acid)] capture assay were used to determine the antioxidant activity of synthetic compounds **2a**-**j** and **3a**-**j**. The results are expressed as IC50 values (concentration in g·mL^−1^). Complexes **3a**, **3b**, **3c**, **3e**, **3g**, and **3i** showed significant DPPH-scavenging activity, as shown in Figure 7. The IC_50_ of these complexes for the control compound BHT was 34.25 g·ml^−1^, while the IC_50_ of the other complexes were 48.11, 47.15, 46.32, 58.17, 57.28, 48.71, and 49.45. The prepared compounds **2a**-**j** and **3a**-**j** had very different IC_50_ for their activities against radical ABTS, ranging from 20.45 to 37.41 g·mL^−1^. Compounds **3e** (35.21 g·ml^−1^), **3i** (31.31 g·ml^−1^), **2d** (35.17 g·ml^−1^) and **2h** (37.41 g·ml^−1^) had the highest activity in the ABTS assay (Figure 7). It should be noted that the reference BHT, a known potent antioxidant molecule, had a free radical ABTS IC_50_ value of 20.38 g·mL. Therefore, the antioxidant activity of Pd complexes hints at their importance in medicine and materials science.

## 4. Conclusion

In conclusion, a series of silver-NHC complexes have been synthesized and studied using various spectroscopic and analytical techniques. The antibacterial properties of each silver-NHC complex were tested against four Gram-negative, three Gram-positive, and one fungal strains. These silver-NHC complexes showed antibacterial activity against bacteria and fungi with MIC values ranging from 6.25 to 100 g/ml. Different substituents on the NHC ligands were found to alter the biological activity of the complex against bacteria. Different nitrogen atom substituents have different effects on antibacterial activity. A bulkier and lipophilic substituent directly attached to the nitrogen atom of the benzmidazol-2-ylidene ligand positively affects antibacterial activity. Therefore, this study will help researchers develop new antibacterial and antifungal drugs with higher potency. [28, 43, 55–57].

## Figures and Tables

**Table 1:** Physical and spectroscopic properties of silver-carbene complexes 3a-3j.

Compounds	Molecular formula	Isolated yield (%)	M.p (°C)	FT-IR *ν*_(CN)_(cm^−1^)	^13^C NMR Ag-C (carbene) (ppm)^a^
**3a**	C_26_H_35_AgClN_3_O	78	236-237	1442	Not observed
**3b**	C_27_H_37_AgClN_3_O	77	243-244	1443	Not observed
**3c**	C_22_H_33_AgBrN_3_O	34	227-228	1443	Not observed
**3d**	C_20_H_29_AgBrN_3_O	70	73-74	1446	Not observed
**3e**	C_22_H_26_AgCl_2_N_3_O	80	227-228	1441	Not observed
**3f**	C_22_H_27_AgClN_3_O	70	214-215	1440	Not observed
**3g**	C_18_H_27_AgClN_3_O_2_	67	114-115	1434	Not observed
**3h**	C_19_H_29_AgClN_3_O_2_	58	250-251	1444	Not observed
**3i**	C_30_H_43_AgBrN_3_O	62	177-178	1449	Not observed
**3j**	C_26_H_35_AgBrN_3_O	62	101-102	1446	Not observed

^[a]^Ag–C(carbene) bond resonance was not observed as a reason for the fluxional behavior of the Ag–NHCs [44]. Bold values represent the number of the synthesized compounds.

**Table 2:** Minimal inhibitory concentration micro mol/L of salts **2** and silver-NHCs complexes **3** against bacterial and fungal strains.

Compounds	Bacteria^[a]^					Fungi^[b]^
	Gram-negative			Gram-positive		
	EC	PA	KP	SA	MRSA	CA
**2a**	21 ± 0.3	42 ± 1.2	22 ± 0.3	11 ± 0.2	13 ± 0.3	8 ± 0.1
**2b**	24 ± 0.4	52 ± 1.3	20 ± 0.7	12 ± 0.5	12 ± 0.4	12 ± 0.3
**2c**	22 ± 0.2	32 ± 0.7	10 ± 0.1	10 ± 0.1	11 ± 0.3	11 ± 0.2
**2d**	39 ± 0.3	36 ± 0.3	25 ± 1.2	12 ± 0.5	13 ± 0.4	12 ± 0.3
**2e**	23 ± 0.6	22 ± 0.2	23 ± 0.7	13 ± 0.4	25 ± 1.2	11 ± 0.2
**2f**	18 ± 0.5	28 ± 0.4	12 ± 0.3	12 ± 0.2	12 ± 0.1	13 ± 0.2
**2g**	29 ± 0.7	37 ± 0.2	22 ± 1.2	11 ± 0.3	21 ± 0.8	23 ± 1.2
**2h**	23 ± 0.2	24 ± 1.1	23 ± 0.7	12 ± 0.3	20 ± 0.8	11 ± 0.4
**2i**	22 ± 0.3	24 ± 1.2	24 ± 0.8	12 ± 0.4	24 ± 0.8	10 ± 0.2
**2j**	18 ± 0.4	33 ± 0.8	25 ± 0.4	11 ± 0.1	13 ± 0.5	9 ± 0.4
**3a**	26 ± 0.7	80 ± 1.8	22 ± 0.6	15 ± 0.3	11 ± 0.2	8 ± 0.6
**3b**	51 ± 0.6	100 ± 1.7	28 ± 1.3	12 ± 0.5	12 ± 0.4	16 ± 0.3
**3c**	26 ± 0.3	51 ± 1.2	14 ± 0.2	14 ± 0.6	13 ± 0.4	14 ± 0.3
**3d**	52 ± 0.8	98 ± 1.8	29 ± 1.3	16 ± 0.5	11 ± 0.4	13 ± 0.3
**3e**	23 ± 0.8	26 ± 1.3	24 ± 0.8	12 ± 0.4	27 ± 1.1	15 ± 0.2
**3f**	26 ± 0.7	52 ± 1.7	19 ± 0.4	11 ± 0.3	13 ± 0.2	14 ± 0.3
**3g**	51 ± 1.3	51 ± 1.6	22 ± 0.7	14 ± 0.5	28 ± 0.6	27 ± 0.4
**3h**	26 ± 0.8	27 ± 0.8	26 ± 0.9	12 ± 0.4	26 ± 0.7	14 ± 0.2
**3i**	27 ± 0.9	24 ± 0.9	24 ± 0.8	17 ± 0.2	27 ± 0.5	13 ± 0.2
**3j**	25 ± 0.7	49 ± 1.5	26 ± 0.9	18 ± 0.6	17 ± 0.2	8 ± 0.1
Ampicillin	18 ± 0.5	—	3 ± 0.2	10 ± 0.2	—	—
Fluconazole	—	—	—	—	—	3.12 ± 0.2

^[a]^EC: *Escherichia coli* (ATCC 25988). PA: *Pseudomonas aeruginosa* (ATCC 27853). KP: *Klebsilla pneumonia* (ATCC 700603). SA: *Staphylococcus aureus* (ATCC 29213). MRSA: *Methicillin-resistant Staphylococcus aureus* (ATCC 43300). ^[b]^CA: *Candida albicans* (ATCC 14053). Bold values represent the number of the synthesized compounds.

## Data Availability

The data used to support the findings of this study are available from the corresponding author upon request.

## Supplementary Materials

Supplementary Materials Supporting information for this article is available with the submitted manuscript; IR, 1H, and 13C NMR spectroscopy and MS of the obtained compounds are available. Figure S1. FT-IR spectrum of silver-carbene complex 2a, Figure S2. 1H NMR spectrum of salt 2a (in CDCl3, 400 MHz, 25°C, TMS), Figure S3. 13C NMR spectrum of salt 2a (in CDCl3, 125 MHz, 25°C, TMS), Figure S4 MS spectra of salt 2a, Figure S5. FT-IR spectrum of salt 2b, Figure S6. 1H NMR spectrum of salt 2b (in CDCl3, 400 MHz, 25°C, TMS), Figure S7. 13C NMR spectrum of salt 2b (in CDCl3, 125 MHz, 25°C, TMS). Figure S8 MS spectra of salt 2b, Figure S9. FT-IR spectrum of salt 2c, Figure S10. 1H NMR spectrum of salt 2ac (in CDCl3, 400 MHz, 25°C, TMS), Figure S11. 13C NMR spectrum of salt 2c (in CDCl3, 125 MHz, 25°C, TMS), Figure S12 MS spectra of salt 2c, Figure S13. FT-IR spectrum of salt 2d, Figure S14. 1H NMR spectrum of salt 2d (in CDCl3, 400 MHz, 25°C, TMS), Figure S15. 13c NMR spectrum of salt 2d (in CDCl3, 100 MHz, 25°C, TMS), Figure S16. MS spectra of salt 2d, Figure S17. FT-IR spectrum of salt 2e,Figure S18. 1H NMR spectrum of salt 2e (in CDCl3, 400 MHz, 25°C, TMS), Figure S19. 13c NMR spectrum of salt 2d (in CDCl3, 100 MHz, 25°C, TMS), Figure S20. MS spectra of salt 2e, Figure S21. FT-IR spectrum of salt 2f, Figure S22. 1H NMR spectrum of salt 2f (in CDCl3, 400 MHz, 25°C, TMS), Figure S23. 13c NMR spectrum of salt 2f (in CDCl3, 100 MHz, 25°C, TMS), Figure S24. MS spectra of salt 2f, Figure S25. FT-IR spectrum of salt 2g, Figure S26. 1H NMR spectrum of salt 2g (in CDCl3, 400 MHz, 25°C, TMS), Figure S27. 13c NMR spectrum of salt 2g (in CDCl3, 100 MHz, 25°C, TMS), Figure S28. MS spectra of salt 2g, Figure S29. FT-IR spectrum of salt 2h, Figure S30. 1H NMR spectrum of salt 2h (in CDCl3, 400 MHz, 25°C, TMS),Figure S31. 13C NMR spectrum of salt 2h(in CDCl3, 100 MHz, 25°C, TMS), Figure S32. MS spectra of 2h, Figure S33. FT-IR spectrum of salt 2i, Figure S34. 1H NMR spectrum of salt 2i (in CDCl3, 400 MHz, 25°C, TMS), Figure S35. 13C NMR spectrum of salt 2i (in CDCl3, 100 MHz, 25°C, TMS), Figure S36. MS spectra of 2i, Figure S37. IR spectra of compound 2j, Figure S38. 1H NMR spectrum of salt 2j (in CDCl3, 400 MHz, 25°C, TMS), Figure S39. 13C NMR spectrum of salt 2j (in CDCl3, 100 MHz, 25°C, TMS), Figure S40. MS spectra of compound 2j, Figure S41. IR spectra of compound 3a, Figure S42. 1H NMR spectrum of salt 3a (in CDCl3, 400 MHz, 25°C, TMS),Figure S43. 13C NMR spectrum of 3a (in CDCl3, 100 MHz, 25°C, TMS),Figure S44. MSspectra of compound 3a, Figure S45. IR spectra of compound 3b, Figure S46. 1HNMR spectra of compound 3b, Figure S47. 13CNMR spectra of compound 3b, Figure S48. MS spectra of compound 3b, Figure S49. I spectra of compound 3c, Figure S50. IR spectra of compound 3c, Figure S51. 13CNMR spectra of compound 3c, Figure S52. MS spectra of compound 3c, Figure S53. IR spectra of compound 3d, Figure S54. 1HNMR spectra of compound 3d, Figure S55. 13CNMR spectra of compound 3d, Figure S56. MS spectra of compound 3d, Figure S57. IR spectra of compound 3e, Figure S58. 1HNMR spectra of compound 3e, Figure S59. 13CNMR spectra of compound 3e, Figure S60. MS spectra of compound 3e, Figure S61. IR spectra of compound 3f, Figure S62. 1HNMR spectra of compound 3f, Figure S63. 13CNMR spectra of compound 3f, Figure S64. MS spectra of compound 3f, Figure S65. IR spectra of compound 3g, Figure S66. 1HNMR spectra of compound 3g, Figure S67. 13CNMR spectra of compound 3g, Figure S68. 13CNMR spectra of compound 3g, Figure S69. IR spectra of compound 3h, Figure S70. 1HNMR spectra of compound 3h, Figure S71. 13CNMR spectra of compound 3h, Figure S72. MS spectra of compound 3h, Figure S73. IR spectra of compound 3i, Figure S74. 1HNMR spectra of compound 3i, Figure S75. 13CNMR spectra of compound 3i, Figure S76. MS spectra of compound 3i, Figure S77. IR spectra of compound 3j, Figure S78. 1HNMR spectra of compound 3j, Figure S79. 13CNMR spectra of compound 3j, Figure S80. MS spectra of compound 3j.
