# Supplementary material for: Synthesis, Characterization, Antimicrobial Properties, and Antioxidant Activities of Silver-N-Heterocyclic Carbene Complexes
Source: Bioinorg Chem Appl. 2023 May 26;2023:3066299. doi: 10.1155/2023/3066299 (PMC10238139; doi:10.1155/2023/3066299)
Supplement: Supplementary Materials — Supporting information for this article is available with the submitted manuscript; IR, 1H, and 13C NMR spectroscopy and MS of the obtained compounds are available. Figure S1. FT-IR spectrum of silver-carbene complex 2a, Figure S2. 1H NMR spectrum of salt 2a (in CDCl3, 400 MHz, 25°C, TMS), Figure S3. 13C NMR spectrum of salt 2a (in CDCl3, 125 MHz, 25°C, TMS), Figure S4 MS spectra of salt 2a, Figure S5. FT-IR spectrum of salt 2b, Figure S6. 1H NMR spectrum of salt 2b (in CDCl3, 400 MHz, 25°C, TMS), Figure S7. 13C NMR spectrum of salt 2b (in CDCl3, 125 MHz, 25°C, TMS). Figure S8 MS spectra of salt 2b, Figure S9. FT-IR spectrum of salt 2c, Figure S10. 1H NMR spectrum of salt 2ac (in CDCl3, 400 MHz, 25°C, TMS), Figure S11. 13C NMR spectrum of salt 2c (in CDCl3, 125 MHz, 25°C, TMS), Figure S12 MS spectra of salt 2c, Figure S13. FT-IR spectrum of salt 2d, Figure S14. 1H NMR spectrum of salt 2d (in CDCl3, 400 MHz, 25°C, TMS), Figure S15. 13c NMR spectrum of salt 2d (in CDCl3, 100 MHz, 25°C, TMS), Figure S16. MS spectra of salt 2d, Figure S17. FT-IR spectrum of salt 2e,Figure S18. 1H NMR spectrum of salt 2e (in CDCl3, 400 MHz, 25°C, TMS), Figure S19. 13c NMR spectrum of salt 2d (in CDCl3, 100 MHz, 25°C, TMS), Figure S20. MS spectra of salt 2e, Figure S21. FT-IR spectrum of salt 2f, Figure S22. 1H NMR spectrum of salt 2f (in CDCl3, 400 MHz, 25°C, TMS), Figure S23. 13c NMR spectrum of salt 2f (in CDCl3, 100 MHz, 25°C, TMS), Figure S24. MS spectra of salt 2f, Figure S25. FT-IR spectrum of salt 2g, Figure S26. 1H NMR spectrum of salt 2g (in CDCl3, 400 MHz, 25°C, TMS), Figure S27. 13c NMR spectrum of salt 2g (in CDCl3, 100 MHz, 25°C, TMS), Figure S28. MS spectra of salt 2g, Figure S29. FT-IR spectrum of salt 2h, Figure S30. 1H NMR spectrum of salt 2h (in CDCl3, 400 MHz, 25°C, TMS),Figure S31. 13C NMR spectrum of salt 2h(in CDCl3, 100 MHz, 25°C, TMS), Figure S32. MS spectra of 2h, Figure S33. FT-IR spectrum of salt 2i, Figure S34. 1H NMR spectrum of salt 2i (in CDCl3, 400 MHz, 25 [file 3066299.f1.docx]

**2a**

**Figure S1**. FT-IR spectrum of silver-carbene complex **2a**

**Figure S2**. ^1^H NMR spectrum of salt **2a** (in CDCl_3_, 400 MHz, 25 °C, TMS).

**Figure S3**. ^13^C NMR spectrum of salt **2a** (in CDCl_3_, 125 MHz, 25 °C, TMS).


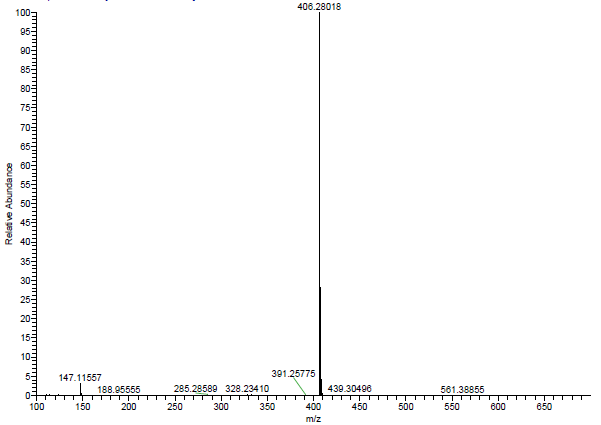


**Figure S4** MS spectra of salt **2a**

**2b**

**Figure S5**. FT-IR spectrum of salt **2b**

**Figure S6. 1H NMR spectrum of salt 2b (in CDCl3, 400 MHz, 25 °C, TMS).**

**Figure S7**. ^13^C NMR spectrum of salt **2b** (in CDCl_3_, 125 MHz, 25 °C, TMS).

**
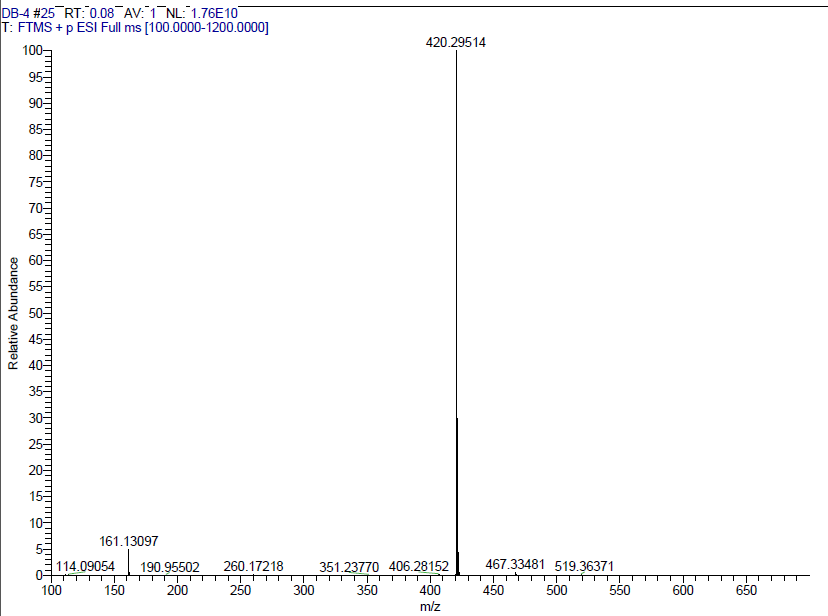
**

**Figure S8** MS spectra of salt **2b**

**2c**

**Figure S9**. FT-IR spectrum of salt **2c**

**Figure S10**. ^1^H NMR spectrum of salt **2ac** (in CDCl_3_, 400 MHz, 25 °C, TMS).

**Figure S11**. ^13^C NMR spectrum of salt **2c** (in CDCl_3_, 125 MHz, 25 °C, TMS).

**
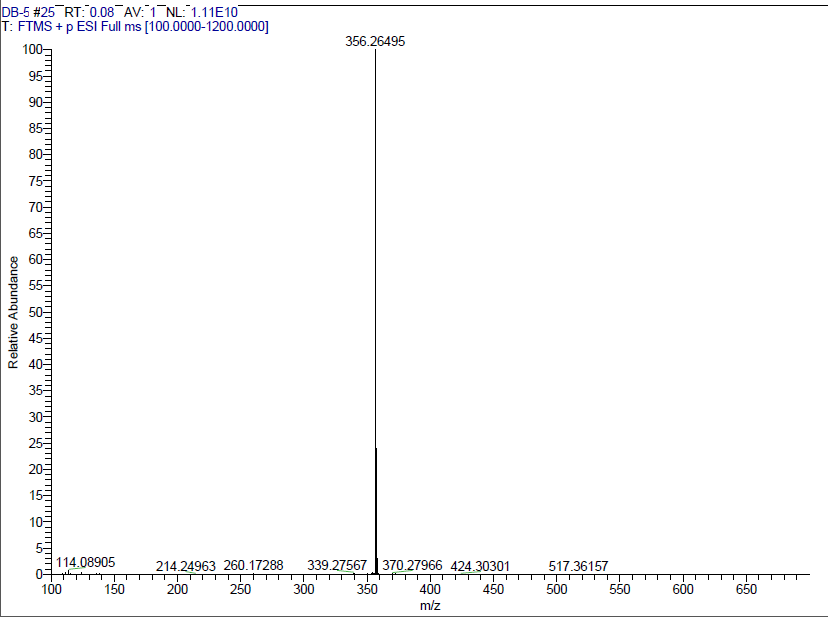
**

**Figure S12 MS spectra of salt 2c**

**2d**

**Figure S13**. FT-IR spectrum of salt **2d**

**Figure S14**. ^1^H NMR spectrum of salt **2d** (in CDCl_3_, 400 MHz, 25 °C, TMS).

**Figure S15**. ^13^c NMR spectrum of salt **2d** (in CDCl_3_, 100 MHz, 25 °C, TMS).

**
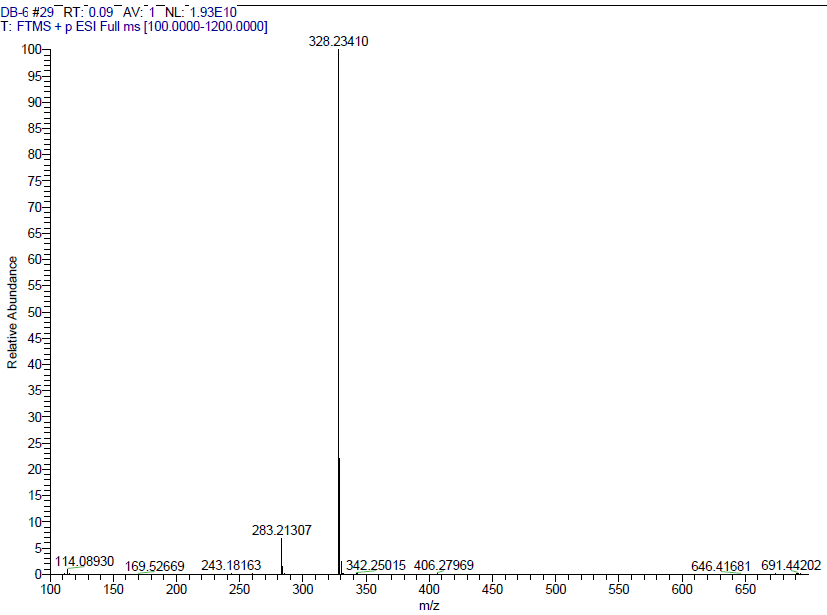
**

**Figure S16**. **MS spectra of salt 2d**

**2e**

**Figure S17**. FT-IR spectrum of salt **2e**

**Figure S18**. ^1^H NMR spectrum of salt **2e** (in CDCl_3_, 400 MHz, 25 °C, TMS).

**Figure S19**. ^13^c NMR spectrum of salt **2d** (in CDCl_3_, 100 MHz, 25 °C, TMS).

**
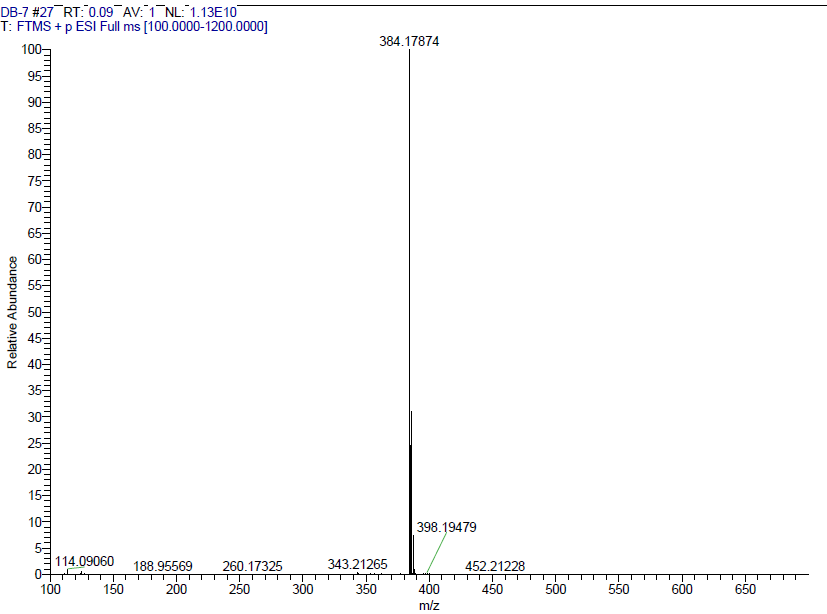
**

**Figure S20**. **MS spectra of salt 2e**

**2f**

**Figure S21**. FT-IR spectrum of salt **2f**

**Figure S22**. ^1^H NMR spectrum of salt **2f** (in CDCl_3_, 400 MHz, 25 °C, TMS).

**Figure S23**. ^13^c NMR spectrum of salt **2f** (in CDCl_3_, 100 MHz, 25 °C, TMS).

**
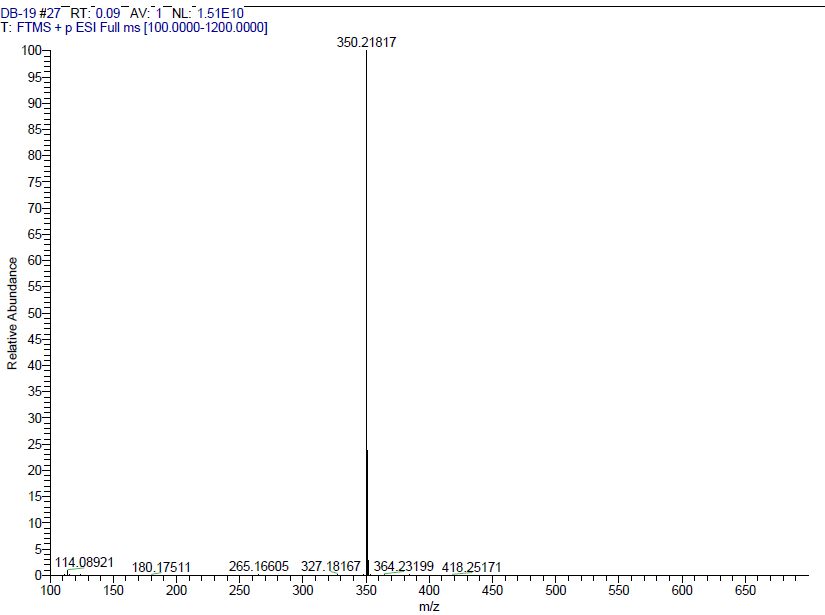
**

**Figure S24**. **MS spectra of salt 2f**

**2g**

**Figure S25**. FT-IR spectrum of salt **2g**

**Figure S26**. ^1^H NMR spectrum of salt **2g**(in CDCl_3_, 400 MHz, 25 °C, TMS).

**Figure S27**. ^13^c NMR spectrum of salt **2g** (in CDCl_3_, 100 MHz, 25 °C, TMS).

**
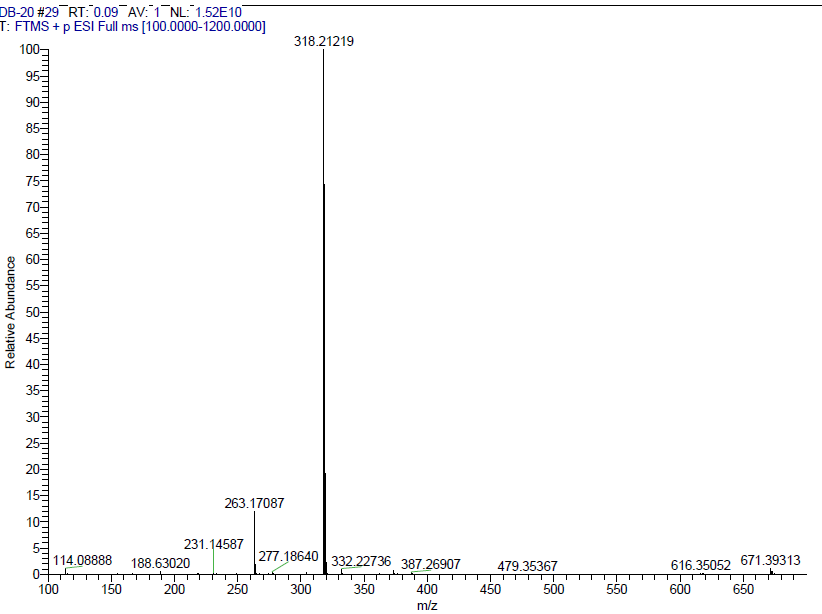
**

**Figure S28**. **MS spectra of salt 2g**

**2h**

**Figure S29**. FT-IR spectrum of salt **2h**

**Figure S30**. ^1^H NMR spectrum of salt **2h**(in CDCl_3_, 400 MHz, 25 °C, TMS).

**Figure S31**. ^13^C NMR spectrum of salt **2h**(in CDCl_3_, 100 MHz, 25 °C, TMS).

**
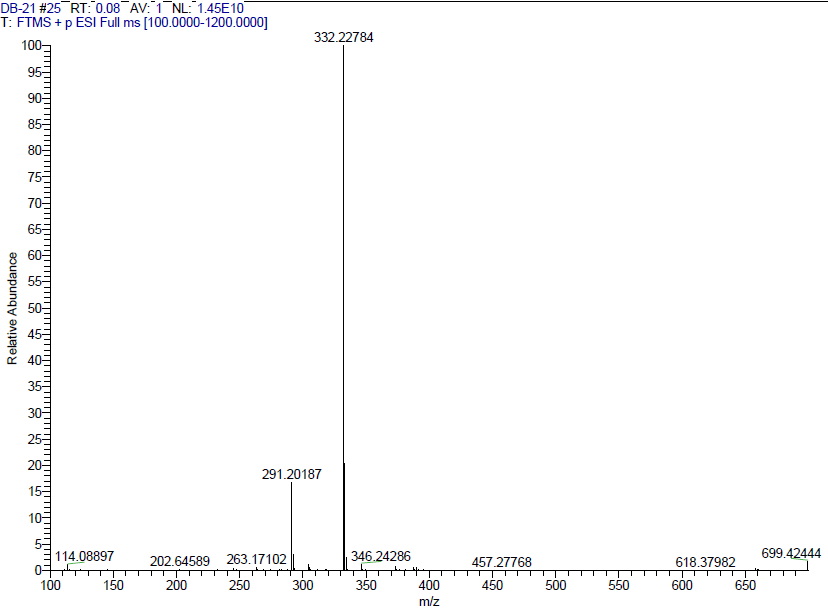
**

**Figure S32**. MS spectra of 2**h**

**2i**

**Figure S33**. FT-IR spectrum of salt **2i**

**Figure S34**. ^1^H NMR spectrum of salt **2i**(in CDCl_3_, 400 MHz, 25 °C, TMS).

**Figure S35**. ^13^C NMR spectrum of salt **2i**(in CDCl_3_, 100 MHz, 25 °C, TMS).

**
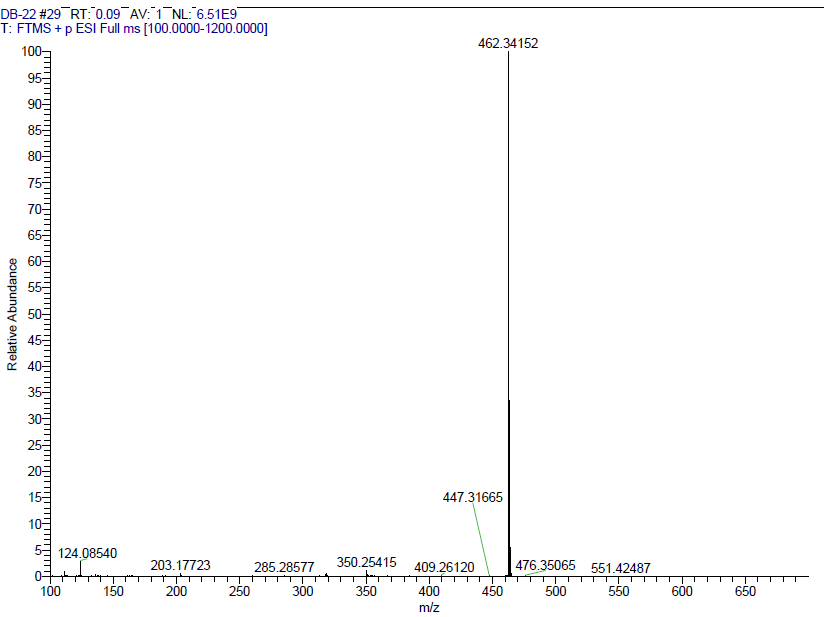
**

**Figure S36**. MS spectra of 2i

**2j**

**Figure S37**. IR spectra of compound 2j

**Figure S38**. ^1^H NMR spectrum of salt **2j**(in CDCl_3_, 400 MHz, 25 °C, TMS).

**Figure S39**. ^1^3C NMR spectrum of salt **2j**(in CDCl_3_, 100 MHz, 25 °C, TMS).

**
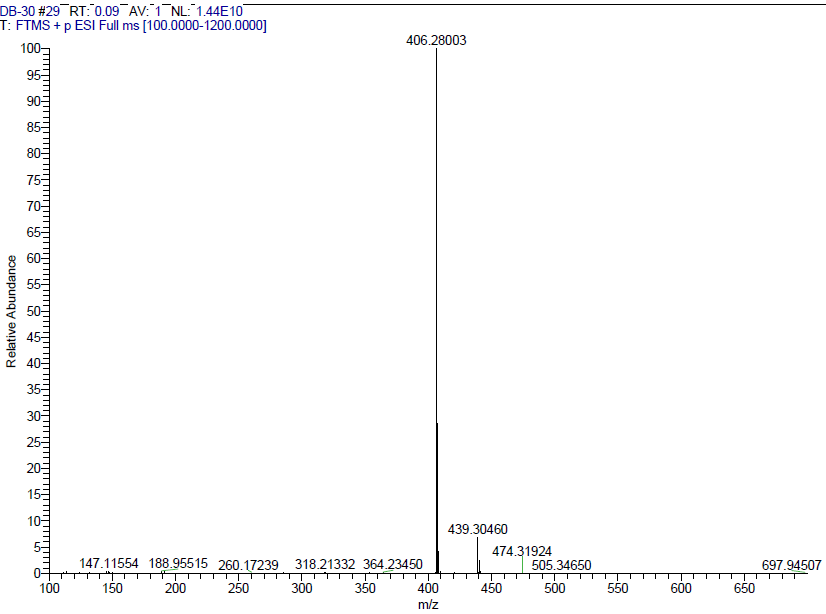
**

**Figure S40**. MS spectra of compound 2j

**3a**

**Figure S41**. IR spectra of compound 3a

**Figure S42**. ^1^H NMR spectrum of salt **3a**(in CDCl_3_, 400 MHz, 25 °C, TMS).

**Figure S43**. ^13^C NMR spectrum of 3a (in CDCl_3_, 100 MHz, 25 °C, TMS).

**
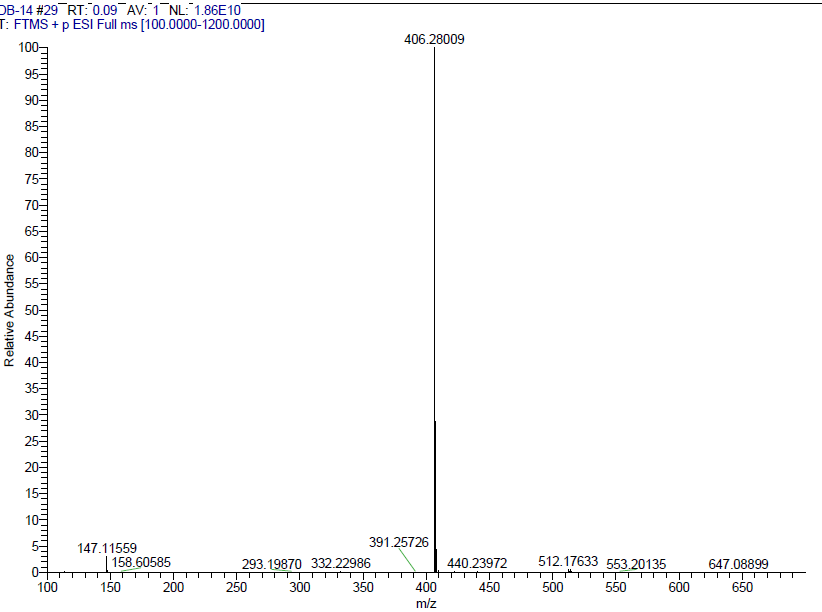
**

**Figure S44**. MSspectra of compound 3a

**3b**

**Figure S45**. IR spectra of compound 3b

**Figure S46**. 1HNMR spectra of compound 3b

**Figure S47**. 13CNMR spectra of compound 3b

**
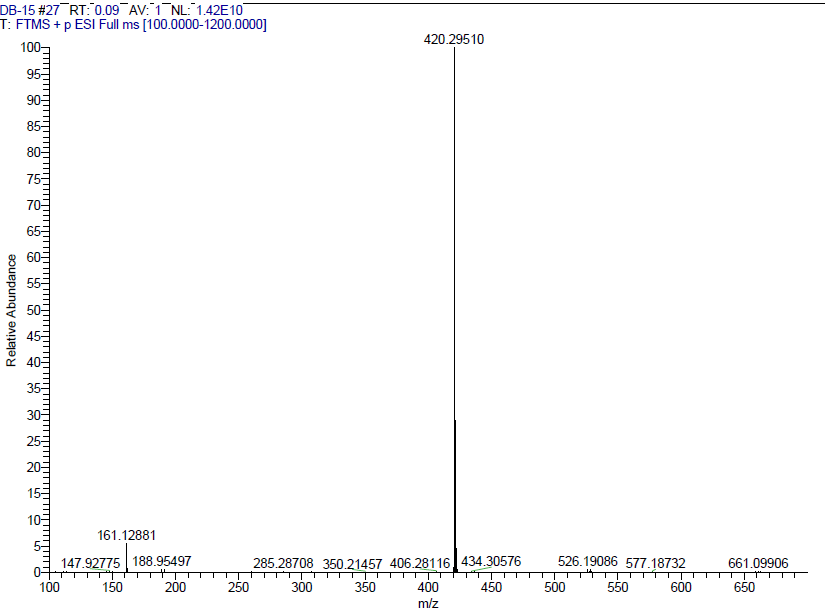
**

**Figure S48**. MS spectra of compound 3b

**3c**

**Figure S49**. IR spectra of compound 3c

**Figure S50**. IR spectra of compound 3c

**Figure S51**. 13CNMR spectra of compound 3c

**
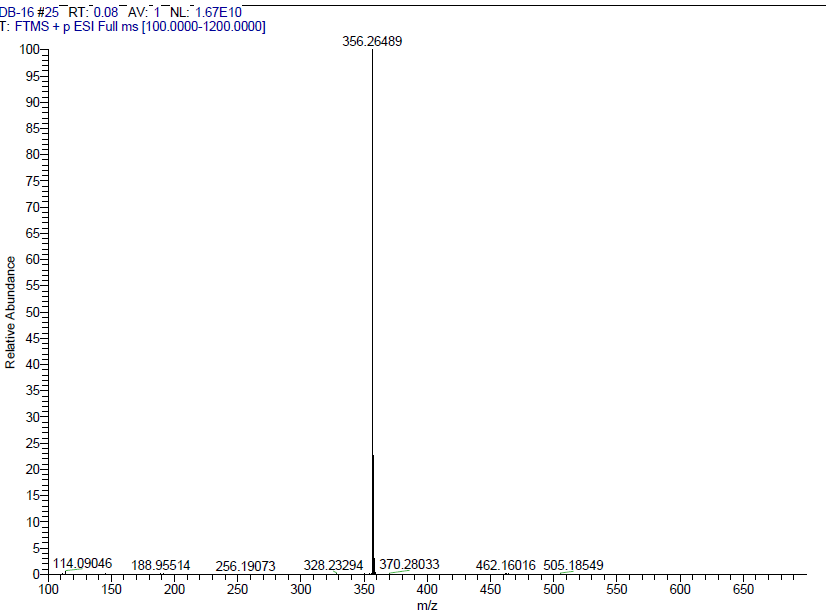
**

**Figure S52**. MS spectra of compound 3c

**3d**

**Figure S53**. IR spectra of compound 3d

**Figure S54**. 1HNMR spectra of compound 3d

**Figure S55**. 13CNMR spectra of compound 3d

**
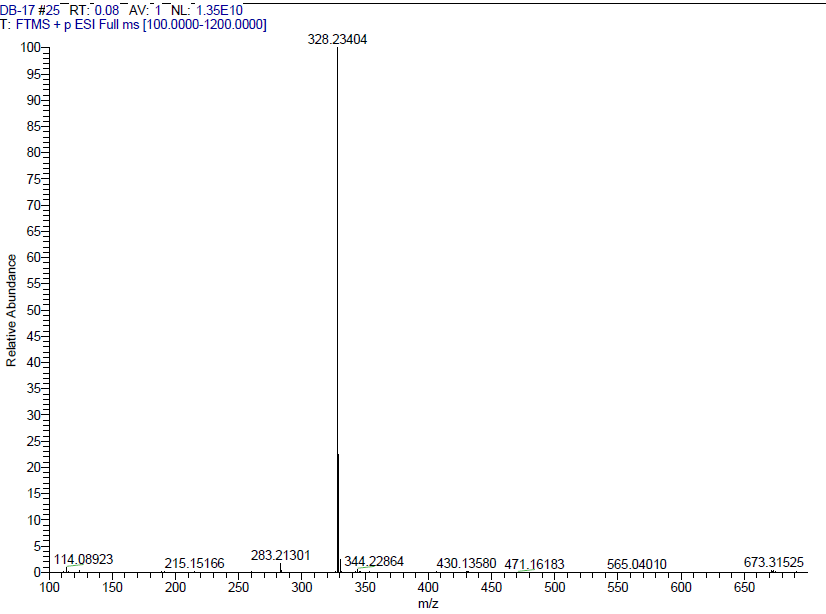
**

**Figure S56**. MS spectra of compound 3d

**3e**

**Figure S57**. IR spectra of compound 3e

**Figure S58**. 1HNMR spectra of compound 3e

**Figure S59**. 13CNMR spectra of compound 3e

**
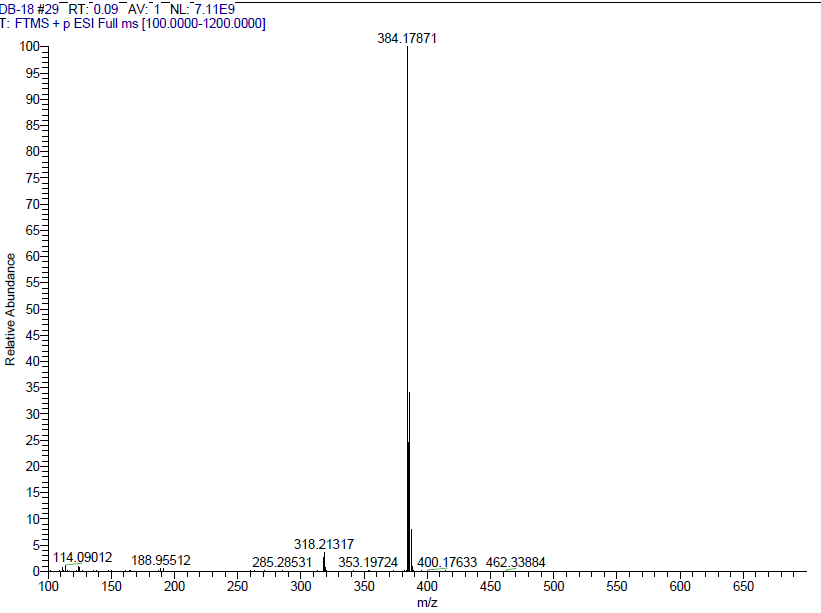
**

**Figure S60**. MS spectra of compound 3e

**3f**

**Figure S61**. IR spectra of compound 3f

**Figure S62**. 1HNMR spectra of compound 3f

**Figure S63**. 13CNMR spectra of compound 3f

**
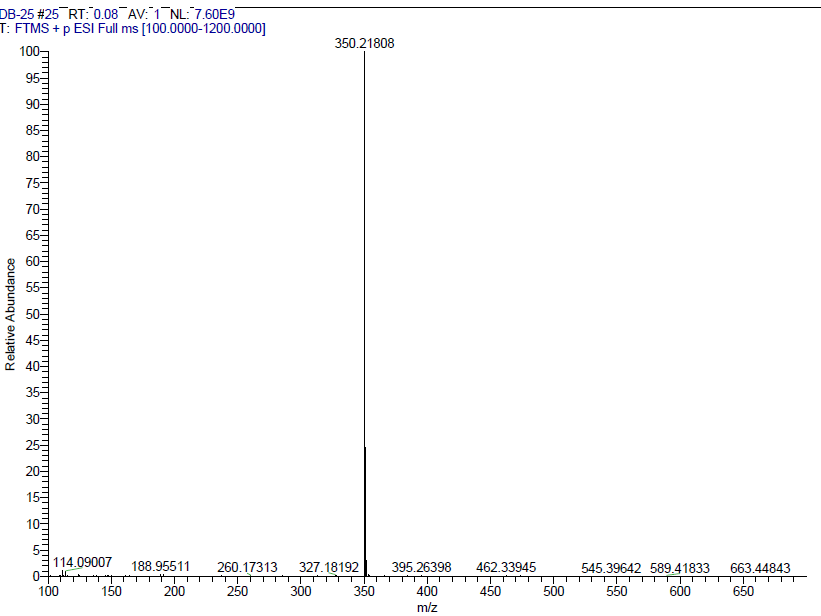
**

**Figure S64**. MS spectra of compound 3f

**3g**

**Figure S65**. IR spectra of compound 3g

**Figure S66**. 1HNMR spectra of compound 3g

**Figure S67**. 13CNMR spectra of compound 3g

**
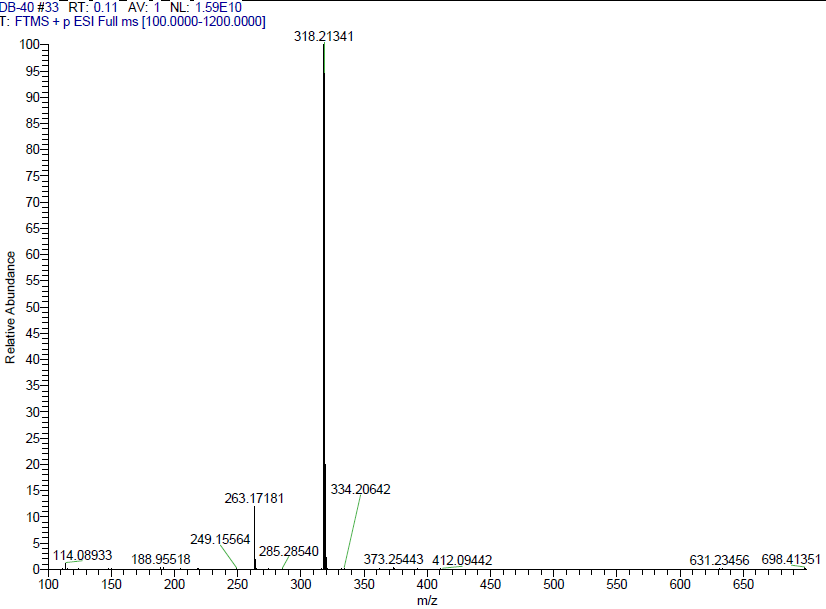
**

**Figure S68**. 13CNMR spectra of compound 3g

**3h**

**Figure S69**. IR spectra of compound 3h

**Figure S70**. 1HNMR spectra of compound 3h

**Figure S71**. 13CNMR spectra of compound 3h

**
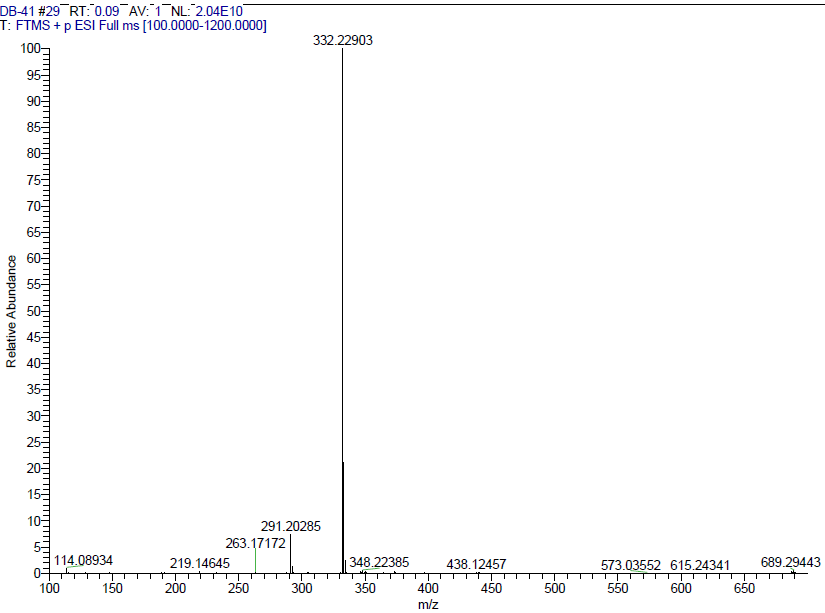
**

**Figure S72**. MS spectra of compound 3h

**3i**

**Figure S73**. IR spectra of compound 3i

**Figure S74**. 1HNMR spectra of compound 3i

**Figure S75**. 13CNMR spectra of compound 3i

**
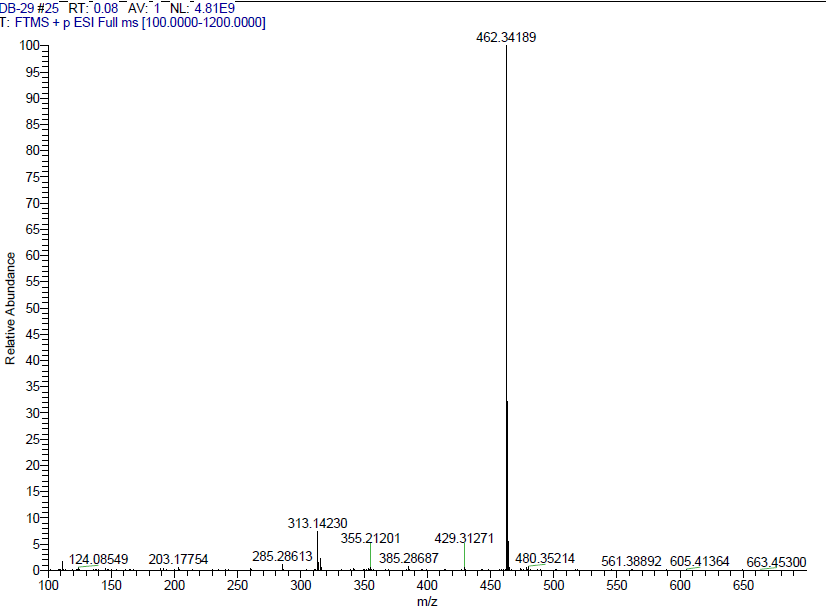
**

**Figure S76**. MS spectra of compound 3i

**3j**

**Figure S77**. IR spectra of compound 3j

**Figure S78**. 1HNMR spectra of compound 3j

**Figure S79**. 13CNMR spectra of compound 3j

**
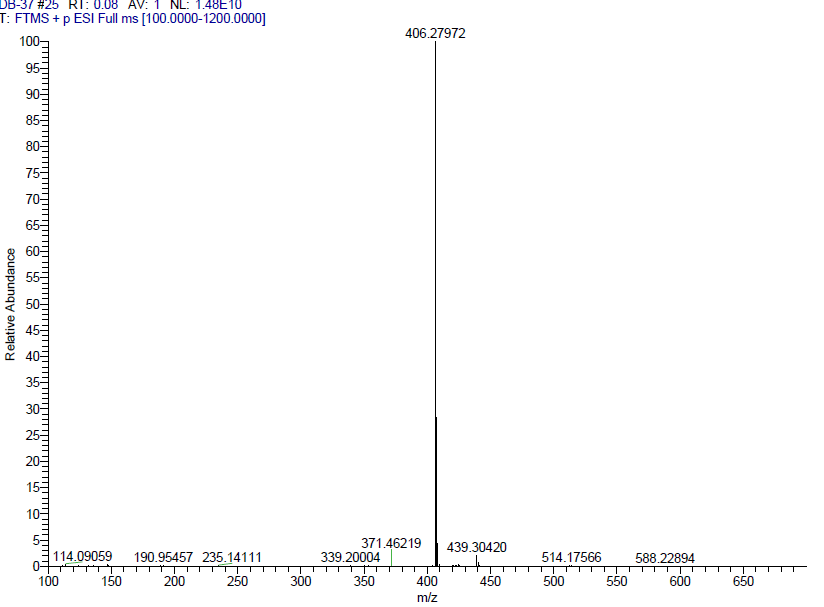
**

**Figure S80**. MS spectra of compound 3j
